# Strong temporal dynamics of QTL action on plant growth progression revealed through high‐throughput phenotyping in canola

**DOI:** 10.1111/pbi.13171

**Published:** 2019-06-12

**Authors:** Dominic Knoch, Amine Abbadi, Fabian Grandke, Rhonda C. Meyer, Birgit Samans, Christian R. Werner, Rod J. Snowdon, Thomas Altmann

**Affiliations:** ^1^ Molecular Genetics/Heterosis Leibniz Institute of Plant Genetics and Crop Plant Research (IPK) Seeland Germany; ^2^ Norddeutsche Pflanzenzucht Innovation GmbH (NPZi) Holtsee Germany; ^3^ Department of Plant Breeding Research Centre for Biosystems Land Use and Nutrition (iFZ) Justus‐Liebig‐University Giessen Giessen Germany; ^4^ Present address: Technische Hochschule Mittelhessen (THM), University of Applied Sciences Fachbereich Gesundheit 35390 Giessen Germany; ^5^ Present address: The Roslin Institute University of Edinburgh Easter Bush Campus Midlothian EH25 9RG UK

**Keywords:** biomass, Brassica, genome‐wide association studies, growth dynamics, high‐throughput phenotyping, vegetative development

## Abstract

A major challenge of plant biology is to unravel the genetic basis of complex traits. We took advantage of recent technical advances in high‐throughput phenotyping in conjunction with genome‐wide association studies to elucidate genotype–phenotype relationships at high temporal resolution. A diverse *Brassica napus* population from a commercial breeding programme was analysed by automated non‐invasive phenotyping. Time‐resolved data for early growth‐related traits, including estimated biovolume, projected leaf area, early plant height and colour uniformity, were established and complemented by fresh and dry weight biomass. Genome‐wide SNP array data provided the framework for genome‐wide association analyses. Using time point data and relative growth rates, multiple robust main effect marker–trait associations for biomass and related traits were detected. Candidate genes involved in meristem development, cell wall modification and transcriptional regulation were detected. Our results demonstrate that early plant growth is a highly complex trait governed by several medium and many small effect loci, most of which act only during short phases. These observations highlight the importance of taking the temporal patterns of QTL/allele actions into account and emphasize the need for detailed time‐resolved analyses to effectively unravel the complex and stage‐specific contributions of genes affecting growth processes that operate at different developmental phases.

## Introduction

Canola/rapeseed (*Brassica napus* L., AACC, 2*n* = 38) is the leading oilseed crop in Canada, Australia, China and Europe and second globally. Its oils have diverse uses, including food, industrial feedstock and as an environmentally friendly renewable energy source (Lu *et al.*, 2011). Early plant growth and biomass formation are crucial traits for productivity and yield, and plant biomass has been shown to be correlated with canola yield at the mature stage (Zhao *et al.*, 2016). Moreover, early‐stage growth is of special importance for young seedlings to provide efficient ground coverage and to avoid competition with weeds in the fields. Today, hybrid varieties dominate the seed market due to their superior early vigour, yield potential and yield stability. Heterosis manifests at a very early stage of seedling development in canola (Basunanda *et al.*, 2010) and plays a key role in field establishment; hence, a better understanding of early plant growth is of great importance for breeding.

The availability of the *Brassica napus* reference genome sequence (Chalhoub *et al.*, 2014) and a 60K SNP genotyping array (Clarke *et al.*, 2016) has enabled genomic studies to greatly improve our understanding of the genetic basis underlying key agronomic traits. Vegetative plant biomass accumulation and growth are under complex genetic control and are strongly influenced by the environment (Shi *et al.*, 2009; Zhao *et al.*, 2016). Thus, dissecting the genetic basis of vegetative plant growth, early plant height and biomass production is of high relevance to fundamental research and to crop improvement strategies. Previous studies applied quantitative trait locus (QTL) mapping and genome‐wide association analyses to identify QTL/alleles for growth (Yong *et al.*, 2015), yield (Luo *et al.*, 2017; Radoev *et al.*, 2008) and yield‐related traits (Cai *et al.*, 2016; Chen *et al.*, 2007; Dong *et al.*, 2018; Yang *et al.*, 2012). In some cases, genes underlying these QTL were also identified (Li *et al.*, 2018; Liu *et al.*, 2015; Zeng *et al.*, 2011). However, most of these studies have focused on single time points, although gene expression patterns are known to change during developmental progression.

Technological advances have resulted in the availability of high‐throughput phenotyping (HTP) offering a non‐invasive, image‐based method to analyse complex traits (Barabaschi *et al.*, 2016). Consequently, many aspects of plant growth and morphological traits have been studied in depth for diverse model and crop plants, including Arabidopsis (Granier *et al.*, 2006; Hartmann *et al.*, 2011; Tisné *et al.*, 2013), maize (Cabrera‐Bosquet *et al.*, 2016; Junker *et al.*, 2015; Zhang *et al.*, 2017), rice (Hairmansis *et al.*, 2014; Schilling *et al.*, 2015; Yang *et al.*, 2014), barley (Honsdorf *et al.*, 2014; Neumann *et al.*, 2015) and rapeseed (Fanourakis *et al.*, 2014; Hatzig *et al.*, 2015; Kjaer and Ottosen, 2015). These new platforms and techniques allow the efficient generation of multiple time point measurements and to assess plant growth and development over time. Furthermore, time is introduced as an additional dimension to association studies.

In Arabidopsis, previous analyses of projected leaf area at 12 different time points, parameters derived from growth models and end‐point biomass data revealed time‐specific and general QTL affecting growth dynamics (Bac‐Molenaar *et al.*, 2015). Similar observations were made regarding temporal patterns of biomass accumulation in barley (Neumann *et al.*, 2017), plant development and height in triticale (Busemeyer *et al.*, 2013; Würschum *et al.*, 2014a; Würschum *et al.*, 2014b) and temporal expression of tiller number in wheat (Ren *et al.*, 2018). Dynamic QTL for plant height and for stress‐responsive and several root traits at different developmental stages was recently reported in upland cotton (Liang *et al.*, 2014; Pauli *et al.*, 2016; Shang *et al.*, 2016). In triticale, genetic dynamics underlying biomass yield were assessed in three developmental stages (Liu *et al.*, 2014). Interestingly, besides detecting QTL active in all stages, some QTL contributed only in one or two of the stages to biomass development. Moreover, a recent study in maize assessed the genetics of growth dynamics at 11 different developmental time points and reported main effect QTL and epistatic interactions with different patterns of expression and reversing allelic effects (Muraya *et al.*, 2017). In *B. napus*, dynamic QTL for plant height were described that showed opposite genetic effects in different periods/stages and experiments (Wang *et al.*, 2015). However, multiple time point analyses to uncover the genetic basis for biomass and growth as dynamic traits have so far not been addressed in canola.

In summary, the studies mentioned above highlight the need to investigate QTL/allele effects by time‐series data to efficiently elucidate growth processes and to detect stage‐specific loci that would likely be missed by analysing single or end‐point data only. Hence, here we investigated a genetically diverse population of 477 spring‐type *B. napus* lines from a canola breeding programme by daily automated high‐throughput phenotyping and performed genome‐wide association analyses throughout an early vegetative phase to address the following questions: (i) Which key genomic regions are associated with growth‐related traits and relative growth rates in the early phase of vegetative plant development? (ii) To what extent do identified regions contribute to trait variance? (iii) Can we resolve dynamic, stage‐specific contributions of loci for early growth by a time course analysis? and (iv) Are we able to nominate candidate genes that might be causal for the observed marker–trait associations?

## Experimental procedures

### Genetic material and plant cultivation conditions

The experimental materials consisted of a total of 477 genotypes (Data S1) from a diverse population of spring‐type *B. napus* canola with double‐low seed quality (low erucic acid, low glucosinolate content). Plants were cultivated under controlled environmental conditions in an incomplete randomized block design (Data S2) in four glasshouse experiments in spring and winter 2014. Data of an additional experiment with a selection of 120 hybrids were included in the calculation of the BLUEs but not in the GWAS, as no array data are available. Experiments were carried out in the IPK phenotyping facility for large plants (Junker *et al.*, 2015) comprising a cultivation, transportation and imaging system with 396 mobile carriers. Each genotype was replicated three times. A container with nine plants comprised one replicate. Four lines were included as checks in higher replication (‘*Achat*’ *n* = 12, ‘*Campino*’ (*CR 3430*) *n* = 12 and the two male sterile testers ‘M1’ and ‘M2’ each *n* = 6 per experiment, respectively) in all cultivations. Plants were grown in large 25‐litre square containers (Bamaplast S.r.l., Massa e Cozzile, Italy) in red substrate 2 (Klasmann‐Deilmann GmbH, Geeste, Germany) to provide enough space for the plants to grow and to avoid pot size effects (Poorter *et al.*, 2012), and covered with a blue rubber mat to facilitate image analysis. A controlled climate regime was applied, on the one hand to mimic natural field‐like conditions and on the other hand to ensure consistency of conditions among the experiments. Temperatures were kept constant with 10 °C (dark phase) and 15 °C (light phase) during the entire growth period, and the natural radiation was supplemented by additional illumination of 205–245 μmol/m^2^/s PAR using SonT Agro high‐pressure sodium lamp. The light period was set to 16‐h light from 06:00 h to 22:00 h. These conditions correspond to a typical early spring in central Europe. Relative air humidity was kept at a minimum of 65%. Initially, nine plants per container were cultivated. To ensure homogenous plant density, two seeds per position were sown, but were thinned to one seedling per position at 5 days after sowing (DAS). Before sowing, seeds were stratified for three days at 4 °C on moist filter paper in Petri dishes to trigger uniform germination. At 14 DAS, four plants per container were sampled to provide enough material for subsequent molecular/biochemical analyses. The remaining five plants were grown until 28 DAS. Watering was performed with an automated balance/watering station by target weight of the containers to maintain 80% field capacity, pH 5.5. Plants were shuffled each day by one row and every second day by one block (11 neighbouring carriers in one row) in the system to minimize position effects.

### High‐throughput plant phenotyping

Over a duration of three weeks (between 6 DAS and 28 DAS), plants were subjected to a daily imaging routine involving automated capturing of top and side view images. Two types of illumination and camera systems in the IPK automated non‐invasive plant phenotyping system for large plants were used as described in Junker *et al. *(2015). Visible light (VIS) and static fluorescence (FLUO) image data were acquired. Each carrier was imaged with two camera systems with four/three side views taken at (0°, 45°, 180° and 225°) from 6 to 13 DAS and (0°, 45° and 135°) from 15 to 27 DAS. Shoot fresh weight (g) was determined on the basis of all five plants by cutting the shoots directly above the ground level and by weighing using a medium‐scale balance at 28 DAS. Dry weight was measured after drying the plant material for 3 days at 80 °C.

### Automated phenotypic data analysis

Automated image analysis was performed using the IPK Integrated Analysis Platform, IAP version 2.07 (Klukas *et al.*, 2014) implementing a customized pipeline combining top and side view images, and including image preprocessing, segmentation and feature extraction. Estimated biovolume, ‘combined geometry vis volume iap (voxel)’, was estimated combining information from top and side view images (Junker *et al.*, 2015). Projected leaf area ‘top geometry vis area (px^2^)’ was derived from VIS top view, early plant height ‘side geometry fluo height (px)’ from FLUO side view, and plant colour uniformity ‘side intensity vis lab a stddev’ from VIS side view images, respectively. Colour uniformity is given as the standard deviation of the *a*‐values in the L*a*b* colour space of the plant pixels. The lower this value, the more uniform is the plant colour. Leaf colour differs between young and old leaves, and therefore, this trait may act as a proxy for the range of maturation stages of leaves within a given plant and thus of its rate of development. Traits represent information obtained from the analysis of whole containers, including nine plants at early and five plants at later stages, respectively.

### Data normalization and statistical analyses

All statistical analyses were performed in R version 3.4.2 software environment for statistical computing (R Core Team, 2018) and graphics and RStudio version 1.1.419. Image‐derived traits were obtained from 6 DAS to 13 DAS and from 15 DAS to 27 DAS. An outlier correction was performed in a combined approach of manual exclusion (carriers with insufficiently germinated plants) and a threshold‐based filtering procedure (median ± 3 standard deviations) for each experiment, day and trait separately. We performed a single‐step analysis of the phenotypic data. Best linear unbiased estimators (BLUEs, Data S3) were estimated in R {lme4} (Bates *et al.*, 2015) based on a linear mixed model for each image‐derived phenotypic trait and each day separately (Eq. 1) or in case of end‐point biomass data (Eq. 2). In the models, *Y* denotes the phenotypic value of a trait for each genotype, *G* represents the fixed effect of the genotype, *E* the random effect of the Experiment, *GxE* the genotype–experiment interaction, *C* the random effect of the included checks, *CxE* the check–experiment interaction, *P* the position in the pot, *PxE* the position–experiment interaction and *e* the residual error (errors were assumed to be normally, independently and identically distributed). Broad‐sense heritabilities (*H* ^2^) for each trait were estimated by Eq. 3, where $\sigma_{G}^{2}$ and $\sigma_{e}^{2}$ denote the variance components of the genotype and the residual variance, respectively, and *n*_0_ the number of experiments or in case of the end‐point biomass data the number of plant replicates per genotype (He *et al.*, 2016; Nakagawa and Schielzeth, 2010). Variance components $\sigma_{G}^{2}$ and $\sigma_{e}^{2}$ were extracted from the mixed linear models (Eq. 1 or Eq. 2) in R {lme4} assuming that all effects were random effects.

(1) Y=G+E+GxE+C+CxE+e

(2) Y=G+E+P+GxE+C+CxE+PxE+e

(3) $$H^{2}=\frac{\sigma_{G}^{2}}{\sigma_{G}^{2}+\frac{1}{n_{0}}\sigma_{e}^{2}}$$

### Calculation of absolute change and relative growth rates

Absolute change rates (ACRs) and relative growth rates (RGRs) were calculated using previously published procedures (Hunt, 1990). Relative growth rates (RGRs) were determined for the estimated biovolume, projected leaf area and early plant height (Eq. 4). To compensate for a potential growth bias due to the applied plant rotation/shift in image acquisition, growth rates were calculated with minute precision, as image acquisition date and time were documented. In addition, absolute change rates (ACRs) were calculated (Eq. 5) for plant colour uniformity. BLUEs for ACR and RGRs were subsequently estimated as described above (Eq. 1).

(4) $$\text{RGR}=\frac{\left(\log_{e}W_{2}-\log_{e}W_{1}\right)}{\left(t_{2}-t_{1}\right)}$$

(5) $$\text{ACR}=\frac{\left(W_{2}-W_{1}\right)}{\left(t_{2}-t_{1}\right)}$$

### Reference genome version and gene annotations

To ensure the unique positioning of as many markers as possible, an enhanced version of the *Brassica napus* cv. Darmor‐*bzh* v4.1 reference genome assembly (Chalhoub *et al.*, 2014) was used, generated by incorporating long read information (NRGene, DeNovoMAGIC™; unpublished data from David Edwards, University of Western Australia) into the pseudomolecules. Transcripts were predicted *de novo* using a MAKER pipeline with AUGUSTUS and SNAP. Subsequently, the transcriptome was annotated by mapping the transcript sequences on: (i) the *B. napus* Darmor‐*bzh* v.4.1, (ii) a concatenated *Brassica* AC‐genome assembly comprising the *B. rapa* v1.5 (Wang *et al.*, 2011) and the *B. oleracea* TO1000 (Parkin *et al.*, 2014), and (iii) the *Arabidopsis thaliana* TAIR10 transcriptomes, respectively, using the basic local alignment search tool (BLAST). Transcripts were counted as hit if they reached a minimum similarity of 80% over 40% of the target transcript. If annotations in multiple genomes were obtained, they were prioritized in the order *B. napus*, *B. oleracea/rapa*, *A. thaliana*. The *B. napus* Darmor‐*bzh* v.4.1, *B. rapa* Chiifu‐401–42 and *B. oleracea* TO1000 transcriptomes were functionally annotated using Blast2GO (Conesa *et al.*, 2005) version 3.0.8 with default settings. The Arabidopsis TAIR transcriptome annotations were downloaded from the TAIR homepage (https://www.arabidopsis.org/).

### Genotype data

All 477 genotypes were genotyped using the *Brassica* Infinium 60k genotyping array (Illumina Inc., San Diego, CA, USA) as described previously by Jan *et al. *(2016). Raw data were initially filtered to exclude SNPs without positional information in the *Brassica rapa* and *Brassica oleracea* genomes. SNP genotypes were called using R and the package {gsrc} (Grandke *et al.*, 2017). To identify copy number variations (CNVs), the SNP positions together with the signal intensity values were used to define blocks of similar intensity. If the blocks' values exceed the applied thresholds, they are classified as deletions or duplications. This set of copy number variations, also generated with the package {gsrc}, complemented the obtained SNP data. Subsequently, probe oligonucleotide sequences were mapped to the enhanced *Brassica napus* cv. Darmor‐*bzh* reference genome assembly using the basic local alignment search tool (BLAST) with 95% similarity over a length of 50 bp. Markers showing multiple BLAST hits in the genome were removed. For genome‐wide association studies (GWAS), SNPs were coded in numerical format (0 = AA, 1 = AB, 2 = BB) using R {GAPIT} (Lipka *et al.*, 2012; Tang *et al.*, 2016). CNVs were included as 0 = normal, 2 = deletion/duplication, whereby the reciprocal events, either duplications or deletions, were treated as missing values. For CNVs, positions were shifted by ±1 bp to avoid identical marker positions. Furthermore, markers with minor allele frequencies (MAF) smaller than 0.01 and markers with more than 10% missing values or more than 25% heterozygous alleles were removed. A total of 16 311 markers comprising 13 201 unique, single‐copy SNPs, 3106 deletions and 4 duplications remaining after filtering were used for analyses (Data S4). Population structure was analysed using the programme STRUCTURE, version 2.3.4 (Pritchard *et al.*, 2000), marker data of all 477 lines and the ‘admixture’ model. Population clustering for *K* = 1–10 was performed with a burn‐in period of 10 000, 10 000 MCMC replications and three iterations per *K*. The lambda parameter was inferred and adjusted to λ = 0.304. The mean Ln probability [L(*K*)] and population clustering for *K* = 2–5 are shown in Figure S9.

### Genome‐wide association studies (GWAS)

Recently, a new method for genome‐wide association studies, FarmCPU (Fixed and random model Circulating Probability Unification), has been proposed by Liu *et al. *(2016a), which controls false positives and effectively reduces false negatives. The method iteratively performs marker tests with pseudoquantitative trait nucleotides (QTNs) as covariates in a fixed‐effects model and optimization on pseudo‐QTNs in a random‐effects model. To some extent, this process is capable to remove the confounding between testing markers and kinship, to prevent overfitting of the model and to control false positives simultaneously. Genome‐wide association study (GWAS) analyses were conducted in R version 3.4.3 {FarmCPU} on BLUEs of the traits of the 477 canola lines using the filtered set of 16 111 numerically coded SNP (*n* = 13 201) and CNV (n = 3110) markers. Analyses were performed in RStudio on a CentOS 7.2 Linux server (HP ProLiant DL580 Gen9 with HP D3600 Array, 4x Intel Xeon E7‐8880v3@2.3 GHz processors, 144 cores, 1TB RAM, 2x480 GB SSD, 2x 600GB SAS, 12x 8TB SAS). As the programme does not allow for missing marker information in the numeric genotype input file, missing data were replaced by the heterozygous allele. Kinship was calculated using the FARM‐CPU algorithm. Principal component analysis was performed on the centred genotype data using the pca function in R {pcaMethods} (Stacklies *et al.*, 2007). The first ten principal components (PCs) were calculated, and the first four PCs were included into the GWAS model to correct for hidden population structure. The maxLoop parameter was increased to 100, and the optimal threshold for *P*‐value selection of the model in the first iteration was estimated by the FarmCPU.P.Threshold function and set to 0.00001 for all traits. Subsequently, *P*‐values of marker–trait associations were adjusted for multiple comparisons using FDR (Benjamini and Hochberg, 1995). Only associations with adjusted *P*‐values smaller 0.1 were considered as statistically significant and used for further analyses. The phenotypic variance explained (PVE%) by a significant marker was estimated in R (Eq. 6). The sum of squares (SS) and residuals (e) were extracted from the ANOVA fitted with a linear model incorporating the phenotypic values and all significant markers in decreasing order of their *P*‐value.

(6) $$\text{PVE}{\%}_{\text{sig}\text{. marker}}=\left({\text{SS}}_{\text{sig}\text{. marker}}/{\text{SS}}_{\text{all sig}\text{. markers}}+e\right)\times 100$$

### LD analysis and candidate gene identification

Pairwise linkage disequilibrium (LD) was analysed for each chromosome in R {LDheatmap} (Shin *et al.*, 2006) for the SNP marker data across all 477 canola lines.

Linkage disequilibrium decay was calculated in R for both subgenomes separately (Hill and Weir, 1988; Marroni *et al.*, 2011; Remington *et al.*, 2001), as larger differences between the A and C subgenome have been reported (Wu *et al.*, 2016). Candidate gene regions were defined as LD blocks harbouring a significant trait‐associated marker in which flanking markers had strong LD (*r* ^2^ > 0.6), and were extended to the left and right unrelated marker, respectively. All genes within the respective LD block were considered for candidate gene identification. For significant markers outside of LD blocks, the 100 kb flanking regions on either side were searched for candidate genes as suggested by Zhou *et al.* (2017a). Candidate genes were prioritized according to their annotation and gene ontology (GO). A comprehensive list of all genes within the intervals and selected candidate genes for all evaluated traits can be found in Data S5.

## Results and discussion

### Capturing growth dynamics by high‐throughput phenotyping

In the present study, a diverse spring‐type canola breeding population consisting of 477 genotypes with ‘double‐low’ seed quality (low erucic acid, low glucosinolate content) was investigated at an early vegetative growth phase. We applied automated high‐throughput phenotyping daily using the previously described IPK phenotyping platform for large plants (Junker *et al.*, 2015) and performed image analysis with our in‐house image analysis pipeline (IAP) to derive estimations of growth‐related traits at multiple time points (Klukas *et al.*, 2014). The IPK systems have previously been shown to be capable of efficiently tracing plant growth in various species including Arabidopsis (Junker *et al.*, 2015; Tschiersch *et al.*, 2017), rice (Schilling *et al.*, 2015), barley (Chen *et al.*, 2014; Neumann *et al.*, 2015), maize (Muraya *et al.*, 2017) and rapeseed (Pommerrenig *et al.*, 2018). Examples of acquired raw plant images are provided in Figure S1. After quality checks, estimates of biovolume, projected leaf area, early plant height as well as colour uniformity were obtained for 21 consecutive time points from 6 to 27 DAS, covering approximately the first growth phase of canola development from completely unfolded cotyledons to four or more unfolded leaves. All four traits showed broad phenotypic variation resulting in medium to high coefficients of variation (Data S3), with highest values for biovolume and lowest values for colour uniformity. Biovolume and projected leaf area displayed exponential increases over time, while early plant height increased in a linear manner. Colour uniformity increased during the first days, but remained at a rather constant level during the later phase (Figure S2a–d). Image‐derived phenotypes were complemented by manually determined end‐point fresh weight (FW) and dry weight (DW) values at 28 DAS (Figure S3). Both fresh weight and dry weight were strongly correlated (*r* = 0.969, Figure S3) and highly correlated with the image‐derived biovolume estimates at the latest time point, with *r* = 0.929 and *r* = 0.926 for FW and DW at 27 DAS, respectively (Data S6). These high correlations indicate that biovolume estimates can serve as a suitable proxy for the actual plant biomass. To assess the repeatability and quality of the phenotypic data, broad‐sense heritabilities (*H* ^2^) were estimated (Figure S4, Data S3). Over the whole experiment, H^2^ for image‐derived phenotypes ranged between 0.528 (early plant height at 15 DAS) and 0. 874 (projected leaf area at 26 DAS). High *H* ^2^ values of 0.895 and 0.878 were also obtained for fresh weight and dry weight, respectively, facilitating the temporal analysis of trait relationships and forming a solid basis for genetic analyses.

### Genomic data, copy number variations and population structure

Genotyping of the 477 lines was performed on the *Brassica* 60k SNP Infinium consortium array (Illumina Inc., San Diego, CA, USA) as described previously by Jan *et al. *(2016). In addition to single nucleotide polymorphisms (SNPs), copy number variation (CNV) and presence–absence variation (PAV) can provide complementary and valuable information, potentially associated with phenotypic changes (Stein *et al.*, 2017). To make use of this additional source of genetic information, SNPs and CNVs and PAVs were called in a combined approach from the array data as previously described (Grandke *et al.*, 2017).

**Figure 1:**
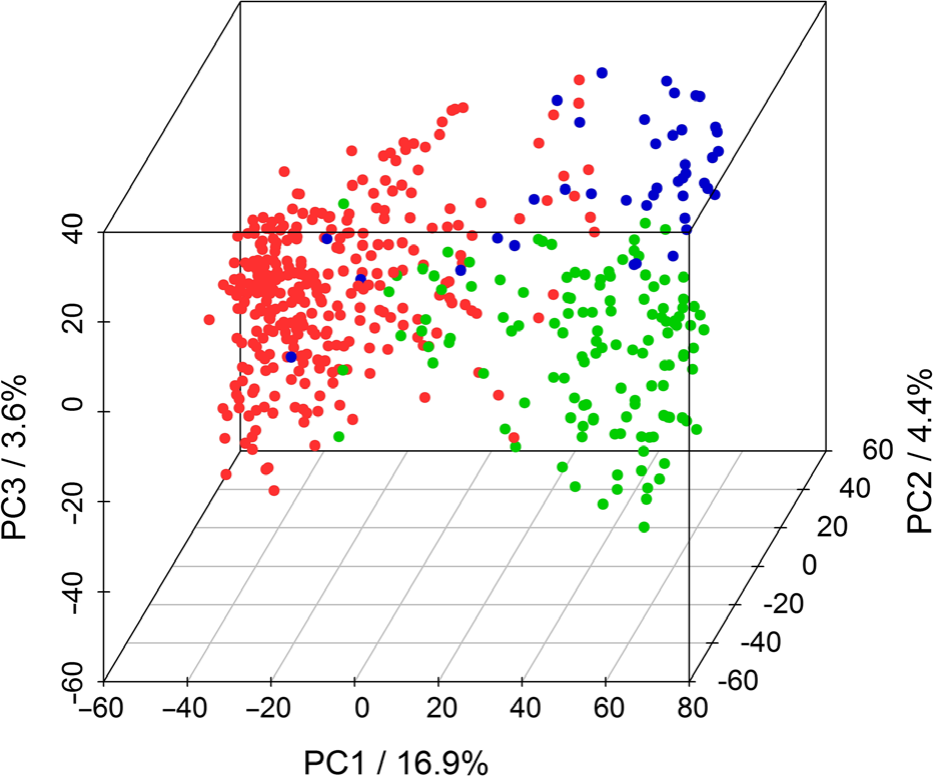
Visualization of breeding pools by principal component analysis (PCA). PCA was performed on 477 canola lines using a panel of 13 201 SNP and 3110 CNV markers. Proportions of explained variance of principal components (PCs) 1, 2 and 3 are indicated on the axes. Different colours correspond to canola breeding pools from which the investigated lines were selected.

A total of 16 311 markers comprising 13 201 unique, single‐copy SNPs, 3106 deletions and four duplications (Data S4) were jointly used in the subsequent genome‐wide association study. Pairwise marker LD matrices (*R* ^2^) were calculated for each chromosome and LD decay derived for both subgenomes (A & C) separately based on the SNP data (Figure S5). In concordance with previous studies (Wu *et al.*, 2016; Zhou *et al.*, 2017b), a faster LD decay was detected in the A subgenome compared to the C subgenome, with half‐decay values of approximately 400 kb and 3.9 Mb determined for the A and C subgenome, respectively. In addition, multiple larger genomic regions of high LD (*R* ^2^ > 0.6) were detected, especially on the C‐subgenome chromosomes (Data S7), pointing to conserved regions preferentially selected during the process of breeding or the presence of larger structural variations within the population compared to the reference genome. A principal component analysis (PCA) of the population was performed using the combined SNP and CNV data sets. The first ten principal components explain a cumulative variance of approx. 40% (PC1: 16.9%, PC2: 4.4%, PC3: 3.6%, PC4: 3.3%, PC5: 2.8%, PC6: 2.0%, PC7: 1.8%, PC8: 1.8%, PC9: 1.6% and PC10: 1.5%). The PCA indicates the existence of population structure (Figure 1), coinciding with the known affiliation of the lines to the three breeding pools within the population (Jan *et al.*, 2016). As breeding pools do not necessarily reflect the genetic structure of the population, we additionally performed a population structure analysis using STRUCTURE (Pritchard *et al.*, 2000). The analysis indicates the presence of two larger population groups and several potential subpopulations (Figure S9). The first three clusters coincide to a substantial degree with the breeding pools, but many individuals show pronounced admixture. As a consequence, the first four principal components, each accounting for more than 3% of the total variance, were included as covariates into the GWAS analysis, as recommended by the developers of the {FarmCPU} R package.

### Predominantly small and medium effect marker–trait associations contribute to variation in growth

BLUEs of image‐derived phenotype data for projected leaf area, estimated biovolume, early plant height and colour uniformity at 21 time points, as well as manually determined biomass (FW and DW) at 28 DAS, were used for genome‐wide association studies using Fixed and random model Circulating Probability Unification, R {FarmCPU} (Liu *et al.*, 2016b). This method features a low rate of false‐positive associations and a fast runtime, which, in combination with parallelization, allows the analysis of multiple phenotypic traits in a reasonable period of time. Although relatively new, the method was already applied successfully in several different studies, for example to identify genetic loci for drought tolerance in maize (Li *et al.*, 2016a), plant height in maize (Hu *et al.*, 2017), salt tolerance in cowpea (Ravelombola *et al.*, 2017), seed traits in soybean (Wang *et al.*, 2018), or tolerance to preharvest sprouting and low falling numbers in wheat (Martinez *et al.*, 2018).

**Figure 2:**
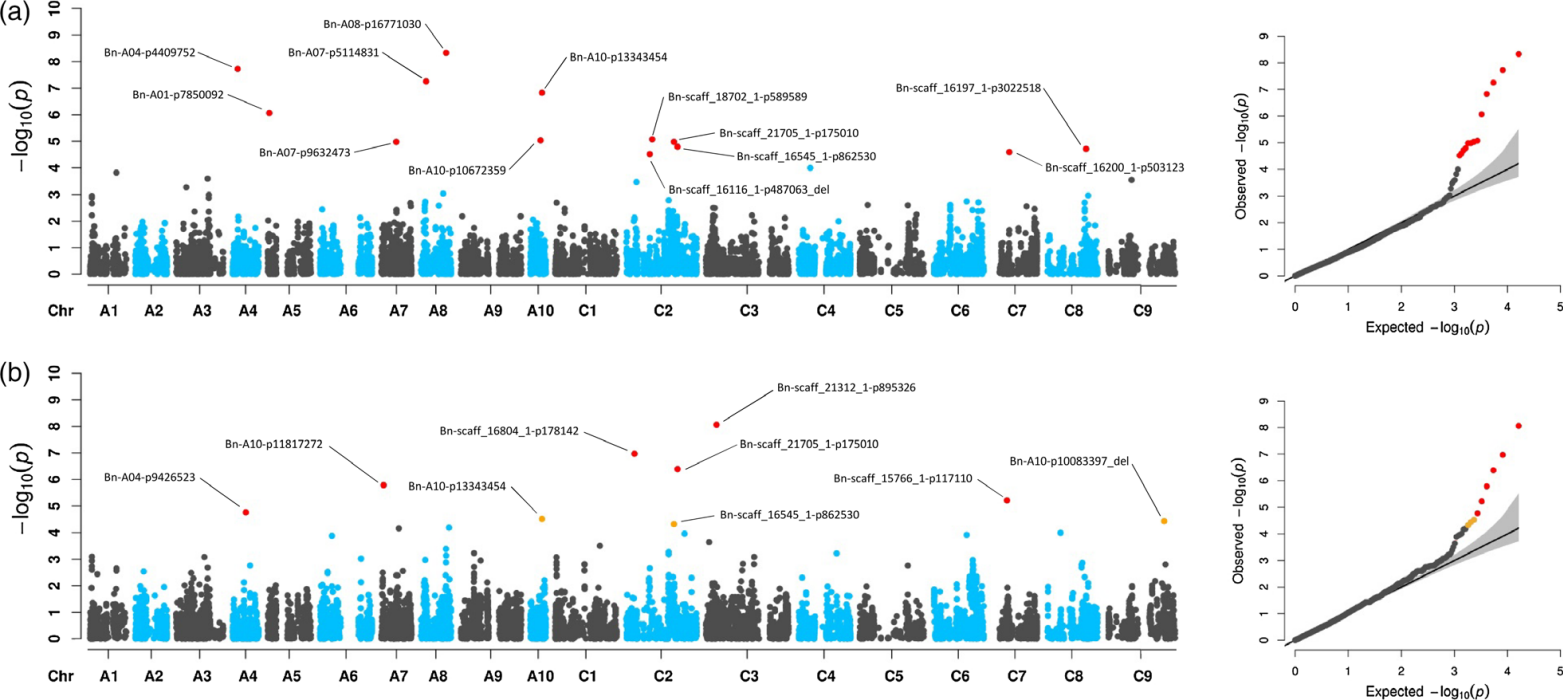
Manhattan and quantile–quantile plots for end‐point vegetative biomass. Genome‐wide marker–trait associations for end‐point biomass determined at 28 DAS. a Manhattan plot (left) and quantile–quantile plot (right) for fresh weight (FW). b Manhattan plot (left) and quantile–quantile plot (right) for dry weight (DW). GWAS was performed in R {FarmCPU} on BLUEs estimated using three replicates (carriers) with five plants each. Significant marker–trait associations (MTAs) are shown with marker IDs. MTAs with *P*‐values_(FDR)_ ≤ 0.05 or 0.1 are indicated by red and orange dots, respectively.

For manually determined biomass, 22 significant marker–trait associations (MTAs) were detected at *P*‐value_(FDR)_ ≤ 0.1 (Figure 2 and Table 1), with thirteen and nine MTAs for fresh weight and dry weight, respectively. Although FW and DW were highly correlated (*r* = 0.969), these traits are not redundant, as similar FW values may break down into different contributions of FW components. Despite the high phenotypic correlation, only three shared MTAs for FW and DW, one on chromosome A10 and two on C02, were identified. We compared the 22 significant MTAs for fresh weight and dry weight to a list of 771 previously described QTL obtained from 13 publications analysing 45 growth, yield and quality‐related traits (Körber *et al.*, 2015, 2016; Li *et al.*, 2016b; Li *et al.*, 2014, 2011, 2017; Liu *et al.*, 2016a; Lu *et al.*, 2016; Luo *et al.*, 2015; Sun *et al.*, 2016; Tang *et al.*, 2015; Wang *et al.*, 2016; Zheng *et al.*, 2017). The marker ‘Bn‐scaff_18702_1‐p589589’ has been shown to be associated with plant height (Tang *et al.*, 2015). Seven other MTAs were in proximity (±500 kb, based on NRGene marker positions) to previously described QTL: ‘Bn‐A04‐p4409752’ close to a QTL for stem dry weight (Lu *et al.*, 2016); ‘Bn‐A10‐p11817272’ close to a QTL for plant height (Sun *et al.*, 2016) and QTL for branch angle (Li *et al.*, 2017); ‘Bn‐A07‐p9632473’ colocalized with a QTL for flowering time (Wang *et al.*, 2016); ‘Bn‐A08‐p16771030’ close to QTL for biomass yield and stem dry weight (Lu *et al.*, 2016) and a QTL for branching angle (Li *et al.*, 2017); ‘Bn‐A10‐p10672359’ in proximity to a QTL for biomass yield, a QTL for stem dry weight (Lu *et al.*, 2016) and a QTL for plant height (Sun *et al.*, 2016); ‘Bn‐A10‐p13343454’ close to another QTL for branching angle (Li *et al.*, 2017); ‘Bn‐scaff_21312_1‐p895326’ close to QTL for stem dry weight, a QTL for biomass yield (Lu *et al.*, 2016) and a QTL for plant height (Li *et al.*, 2016b).

**Table 1:** Information about markers associated with end‐point biomass

Trait	Marker_ID	Chromosome	Position (bp)	MAF	*P*‐value	*P*‐value _(FDR)_	Effect	PVE%*
Fresh weight	Bn‐A04‐p4409752	A04	5 462 587	0.4937	1.89E‐08	0.0002	1.0762	8.64
Fresh weight	Bn‐A01‐p7850092	A05	1 595 585	0.0430	8.62E‐07	0.0028	−2.6757	4.01
Fresh weight	Bn‐A07‐p9632473	A07	15 644 870	0.2715	1.05E‐05	0.0191	−0.9127	5.07
Fresh weight	Bn‐A07‐p5114831	A08	5 664 005	0.3637	5.54E‐08	0.0003	0.9170	1.75
Fresh weight	Bn‐A08‐p16771030	A08	26 455 071	0.2966	4.72E‐09	0.0001	−1.2357	1.37
Fresh weight	Bn‐A10‐p10672359	A10	10 601 845	0.3019	9.28E‐06	0.0191	0.8144	0.02
Fresh weight	Bn‐A10‐p13343454	A10	12 120 357	0.2117	1.48E‐07	0.0006	−1.1502	2.16
Fresh weight	Bn‐scaff_16116_1‐p487063_del	C02	25 078 453	0.0629	3.01E‐05	0.0377	−0.8749	0.33
Fresh weight	Bn‐scaff_18702_1‐p589589	C02	27 593 710	0.0639	8.45E‐06	0.0191	−1.4439	1.21
Fresh weight	Bn‐scaff_16545_1‐p862530†	C02	50 263 120	0.4874	1.05E‐05	0.0191	−0.8823	1.51
Fresh weight	Bn‐scaff_21705_1‐p175010†	C02	54 034 064	0.3344	1.60E‐05	0.0261	0.8604	1.33
Fresh weight	Bn‐scaff_16200_1‐p503123	C07	17 183 655	0.3176	2.52E‐05	0.0342	1.5186	0.57
Fresh weight	Bn‐scaff_16197_1‐p3022518	C08	41 613 071	0.2809	1.92E‐05	0.0285	0.7434	0.44
Dry weight	Bn‐A04‐p9426523	A04	13 935 829	0.1908	1.71E‐05	0.0466	−0.0705	2.11
Dry weight	Bn‐A10‐p11817272	A07	2 411 921	0.2002	1.63E‐06	0.0066	−0.0755	4.85
Dry weight	Bn‐A10‐p13343454	A10	12 120 357	0.2117	3.05E‐05	0.0709	−0.0623	3.56
Dry weight	Bn‐scaff_16804_1‐p178142	C02	9 108 149	0.1122	1.07E‐07	0.0009	−0.1186	5.08
Dry weight	Bn‐scaff_16545_1‐p862530†	C02	50 263 120	0.4874	4.81E‐05	0.0872	−0.0411	0.00
Dry weight	Bn‐scaff_21705_1‐p175010†	C02	54 034 064	0.3344	4.03E‐07	0.0022	0.0736	1.48
Dry weight	Bn‐scaff_21312_1‐p895326	C03	11 220 963	0.0398	8.64E‐09	0.0001	0.2735	5.91
Dry weight	Bn‐scaff_15766_1‐p117110	C07	14 697 010	0.2904	6.13E‐06	0.0200	0.1230	1.47
Dry weight	Bn‐A10‐p10083397_del	C09	59 994 601	0.0273	3.70E‐05	0.0755	0.1283	0.89

* Estimated percentage of phenotypic variance explained by the marker.

† Common MTAs shared between fresh weight and dry weight.

Genome‐wide association analyses performed for data measured at all 21 time points with the moderate threshold (*P*‐value_(FDR)_ ≤ 0.1) revealed a total of 787 MTAs, including 191 associations for estimated biovolume, 200 MTAs for projected leaf area, 182 MTAs for early plant height and 192 MTAs for colour uniformity, respectively. There were no substantial differences in the number of associations between the A and the C subgenomes. The majority of detected associations could be attributed to unique, single‐copy SNP markers (84% of all associations). A substantial number of CNVs (deletions and duplications) also showed trait associations independently of the two SNP alleles (Grandke *et al.*, 2017; Mason *et al.*, 2017). In particular, segmental deletions caused by widespread homoeologous exchanges (Hurgobin *et al.*, 2018; Samans *et al.*, 2017) were associated with trait variation as previously described (Hatzig *et al.*, 2018; Qian *et al.*, 2016; Schiessl *et al.*, 2017; Stein *et al.*, 2017). To reduce the list to robust candidate regions, detected MTAs were further filtered to retain only loci showing significant associations for at least three consecutive time points (Data S8). Most of the detected MTAs explained only a small percentage of phenotypic variance (<5 PVE%, Figure S6) and were randomly distributed over the subgenomes. Only 40 (3.8%) marker–trait associations with larger effects (>5 PVE%) were detected, for example Bn‐A04‐p4409752 explaining up to 8.64% PVE of biomass (fresh weight). These findings strengthen the hypothesis that biomass accumulation and growth‐related traits are mostly governed by small effect loci and their interactions.

### Dynamic genetic patterns and time interval‐specific QTL for early vegetative growth

**Figure 3:**
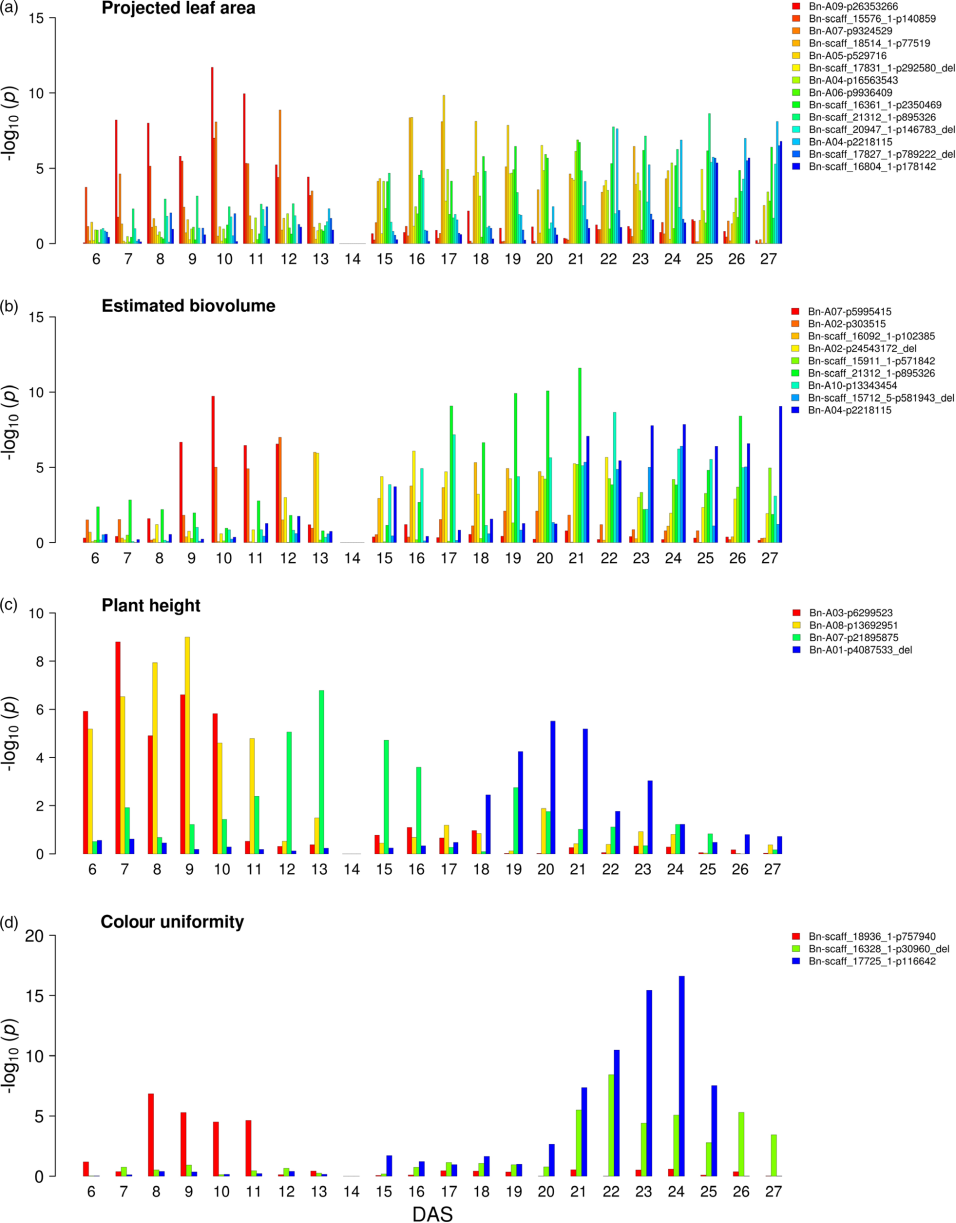
Dynamic associations detectable during cultivation from 6 to 27 DAS. GWAS was performed on BLUEs of a projected leaf area, b estimated biovolume, c early plant height and d plant colour uniformity in R/package {FarmCPU}. Different colours indicate markers with *P*‐value_(FDR)_ ≤0.1 at three consecutive days, with the colour gradient corresponding to the temporal pattern. DAS denotes days after sowing. BLUEs were estimated using three replicates (carriers) with nine and five plants for 6 to 13 DAS and 15 to 27 DAS, respectively. No data were recorded at 14 DAS due to sampling of shoot material.

The time‐resolved design of the phenotyping experiments enabled us to track the effects of individual markers over the course of 21 days of early growth between 6 and 27 DAS. Previous studies in Arabidopsis (Bac‐Molenaar *et al.*, 2015) and maize (Muraya *et al.*, 2017) also addressed the dynamics of growth, but few studies provided such a high temporal resolution at a daily basis. Markers that displayed sequentially significant association with our measured phenotypic traits for multiple consecutive time points were evaluated in more detail. In summary, 14, 9, 4 and 3 MTAs for projected leaf area, estimated biovolume, early plant height and colour uniformity were detected to be significant at three consecutive days, respectively (Figure 3, Data S8). In accordance with the theory of developmental genetics that genes are expressed selectively at different developmental stages, our data indicate that plant growth is the cumulative result of the interaction of various different genes and that the contributing sets of growth factors change during plant development. In contrast to a previous study in Arabidopsis, which revealed time‐specific and general QTL affecting growth dynamics (Bac‐Molenaar *et al.*, 2015), in the present study in canola only time‐specific associations were detected. The longest interval of significance was found for marker ‘Bn‐scaff_16361_1‐p2350469’ on chromosome C08 associated with projected leaf area between 16 and 27 DAS. The nature of these dynamic, time‐specific patterns with their phenotypic plasticity suggests that they are under the control of dynamic genetic regulation. The beneficial effect of an allele of an early QTL might lose its benefit with progression of development and another allele of a later QTL might take up the beneficial effect. Remarkably, the same marker, ‘Bn‐scaff_16804_1‐p178142’ on chromosome C02, was found to be associated with both, the projected leaf area at 25–27 DAS and end‐point dry weight. Many associations with effects at earlier time points would likely not have been identified if biomass‐associated traits had only been evaluated as integrated effects at the end of the experiment. As a result, underlying genes might not be uncovered or the genetic value of the loci might be underestimated.

**Figure 4:**
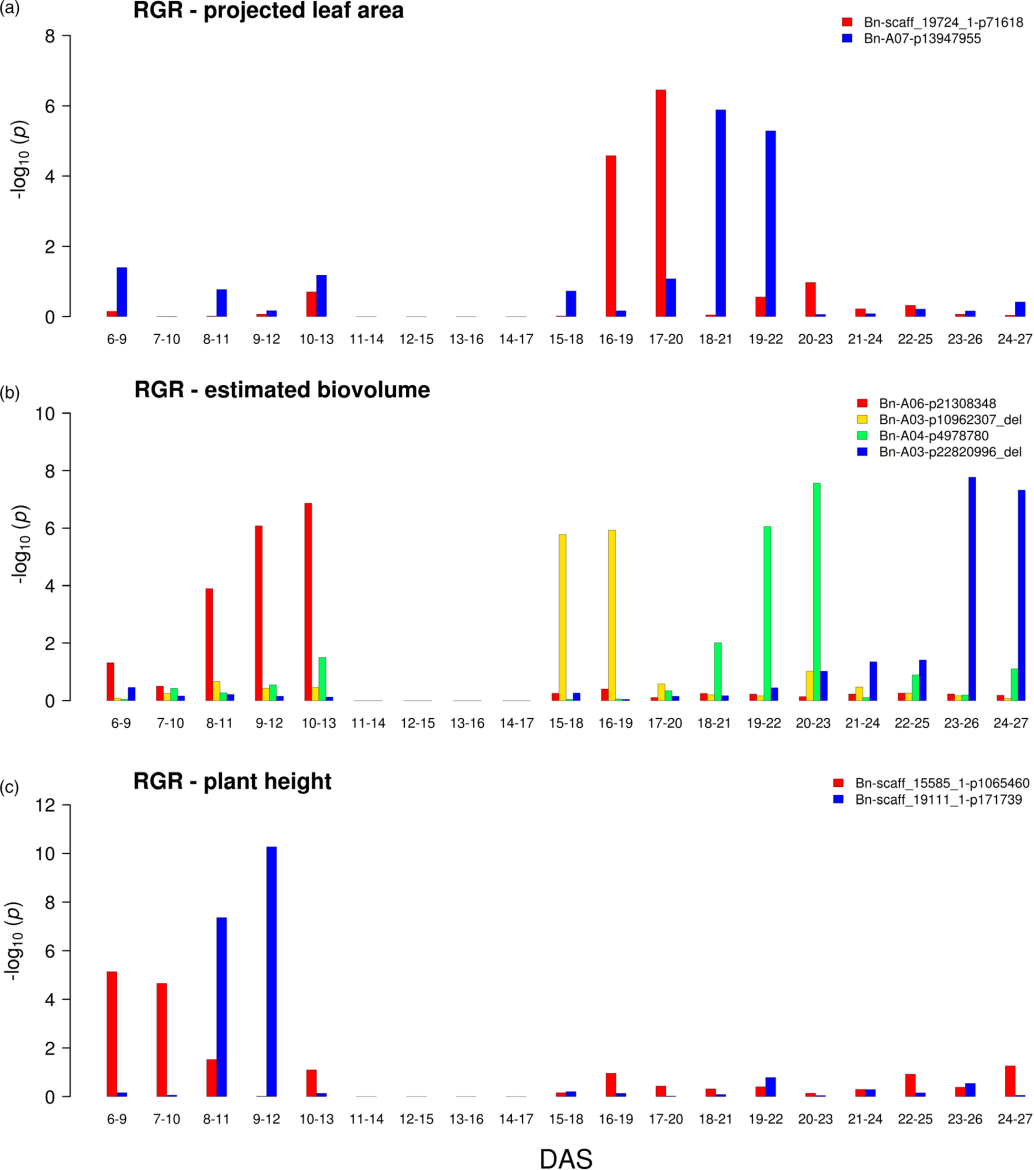
Dynamic associations detectable for relative growth rates. GWAS was performed on BLUEs of a relative growth rates for projected leaf area, b relative growth rates for estimated biovolume and c relative growth rates for early plant height in R/package {FarmCPU}. Different colours indicate markers with *P*‐value_(FDR)_ ≤0.1 at two consecutive intervals. DAS denotes days after sowing. BLUEs were estimated using three replicates (carriers) with nine and five plants for 6 to 13 DAS and 15 to 27 DAS, respectively. No data were recorded at 14 DAS due to sampling of shoot material.

To further address the dynamic nature of these traits, relative growth rates (RGRs) for projected leaf area, estimated biovolume and early plant height, as well as absolute change rates (ACRs), for colour uniformity were calculated over 15 intervals of three day durations to integrate the effects over longer periods (Figure S2e,f). Highest relative growth rates, especially for plant height, were detectable in the beginning of the cultivation and show a decreasing trend over time attributed either to an actual decrease in growth or to a bias due to overlapping leaves. Absolute change rates for colour uniformity were more stable than the relative growth rates during development. Growth rates were subsequently mapped with the same approach as the single time point data. It is remarkable to note that (beyond achievements in previous studies) GWAS was successfully applied here to RGR traits of multiple successive time intervals resulting in the detection of a total of 268 significant associations, with 100 MTAs for biovolume RGRs, 76 MTAs for leaf area RGRs, 73 MTAs for plant height RGRs and 19 MTAs for the colour uniformity changes detected for individual intervals. This can be attributed to the statistical power achieved in the present study through the large dimension and the setup of the experiments assessing each of the 477 analysed genotypes replicated in three of the four large‐scale glasshouse experiments performed under controlled environmental conditions with nine (6 to 13 DAS) or five (15 to 27 DAS) individuals, respectively, per replicate. To focus on particularly robust MTAs, the growth rate associations were further filtered for at least two consecutive significant intervals, as it has been done previously for the absolute trait values at individual time points. For colour uniformity ACRs, no consecutive significant associations were found. Two MTAs for leaf area RGRs at intermediate growth intervals, four MTAs for biovolume RGRs distributed evenly over the entire examined growth period and two MTAs for plant height at a very early phase were detected (Figure 4). The substantially lower number of RGR MTAs active at two consecutive intervals vs. the total number of RGR MTAs may indicate that the majority of effects are restricted to very narrow time windows. Since RGR MTAs address the acute action of the genetic loci at the assessed time point, while the MTAs of absolute trait values reflect the cumulative effects of the loci that happened during the entire growth period up to the time point of measurement, it is not surprising that the number of detected RGR MTAs is generally lower than the number of MTAs of absolute trait MTAs and that there is only minor overlap between the MTAs of the two types of traits.

A recent study analysed the genetic architecture of biomass accumulation in spring barley (Neumann *et al.*, 2017) by image analysis and described temporal patterns similar to the findings for absolute trait MTAs in the present study. Muraya *et al. *(2017) detected MTA effects on RGR for a subset of the strongest absolute trait MTAs and described the reversal of allelic effects over time for markers associated with relative growth rates. Similar observations were made in the present study on canola: allelic effects of loci did not only increase and decrease with time, tending to diminish after a certain interval, but for a substantial fraction of MTAs (16/30 for absolute trait MTAs and even 8/8 for RGR MTAs) allele effects reversed over time (Figures S7 and S8).

As most dynamic growth/biomass‐associated QTL actions tended to persist for periods of only a few days during early growth (a particularly remarkable pattern was observed for the RGR of estimated biovolume; Figure 4b), it might be hypothesized that these QTL are associated with the initiation or development of new leaves. Manual analysis of leaf number for a subset of 30 lines at the different time points indicated that new leaves emerge on average in three‐ to four‐day intervals, coinciding with the observed pattern of dynamic growth QTL. To verify this initial observation, more in‐depth analyses will be necessary that will require robust high‐throughput quantification of leaf number in the acquired images. While promising advances in image analyses have been achieved in this direction, for example by ‘CVPPP challenges’ (Pape and Klukas, 2015; Scharr *et al.*, 2016), further developments will be necessary to use automated image analyses towards this goal. If the hypothesis of different QTL being involved in initiation and development of successive leaves can be supported, it indicates the exciting possibility that formation of each leaf (or more generally every organ) may be controlled by a distinct genetic programme triggered through certain leaf‐specific loci.

### Shared associations and novel candidate genes for growth dynamics

The purpose of our study was to reveal dynamic growth QTL patterns by a time‐resolved association analysis. Our findings highlight the need for stage‐specific investigations in future studies to identify genes operating at different developmental phases. Muraya *et al. *(2017) proposed that genes corresponding to dynamic QTL are either selectively expressed at different growth stages or their functions are required or growth‐limiting only in certain developmental phases.

Among the 36 identified markers displaying temporal dynamic patterns, nine were shared between different traits. In particular, ‘Bn‐A10‐p13343454’ showed association with projected leaf area, estimated biovolume and early plant height, as well as with fresh weight and dry weight. The marker ‘Bn‐scaff_21312_1‐p895326’ was associated with projected leaf area, biovolume and dry weight, while ‘Bn‐scaff_16804_1‐p178142’ was shared between projected leaf area and dry weight. The other six markers: ‘Bn‐A02‐p24543172_del’, ‘Bn‐A04‐p2218115’, ‘Bn‐scaff_15911_1‐p571842’, ‘Bn‐scaff_16361_1‐p2350469’, ‘Bn‐scaff_17831_1‐p292580_del’ and ‘Bn‐scaff_20947_1‐p146783_del’ were associated with both projected leaf area and biovolume.

**Figure 5:**
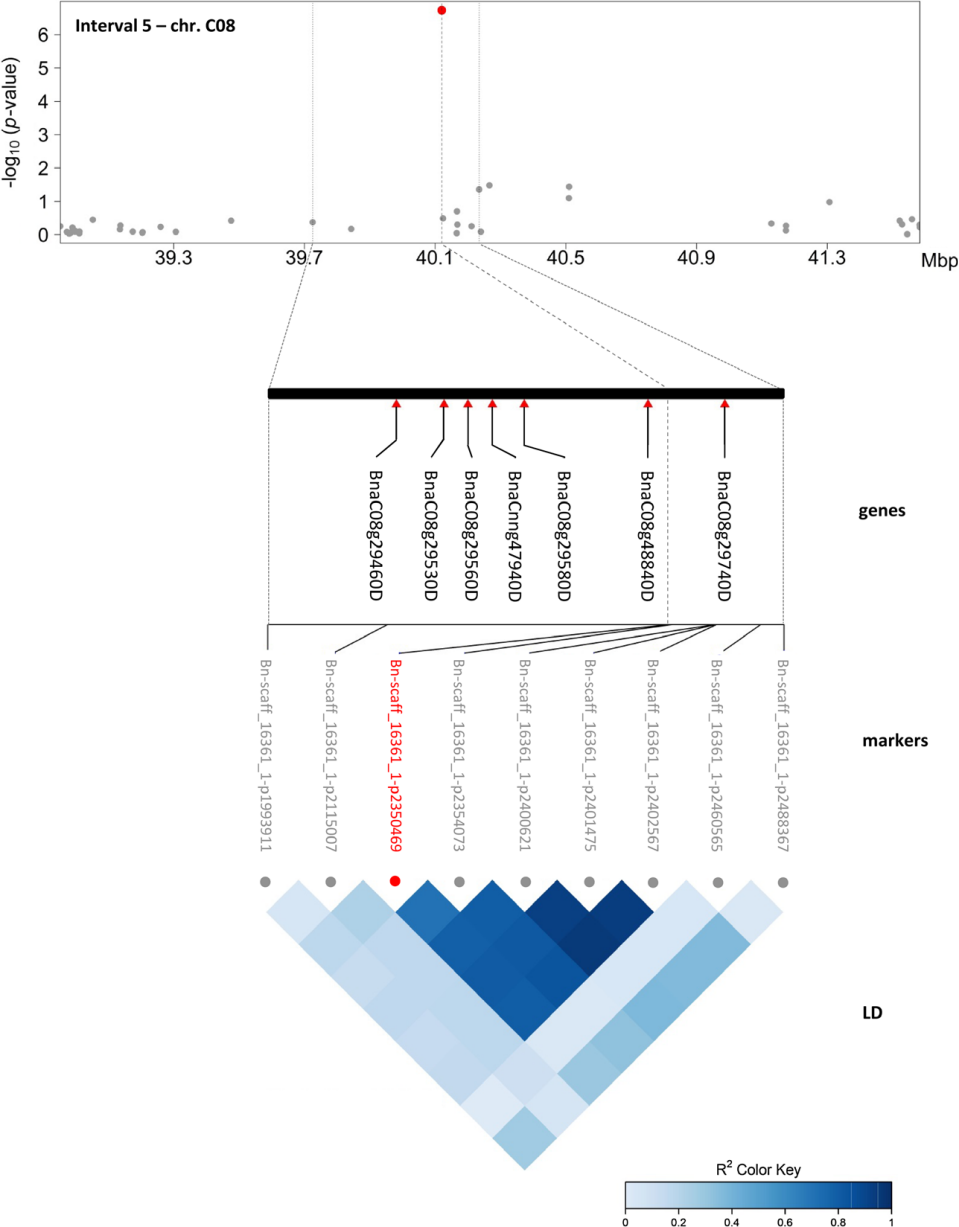
Manhattan plot for a representative MTA in the candidate region 5 on Chr. C08 with selected candidate genes and correlations between markers. The Manhattan plot describes genome‐wide marker–trait associations for the candidate region 5 on chromosome C08. The trait ‘projected leaf area at 21 DAS’ is shown as a representative trait for the 14 traits associated with the marker ‘Bn‐scaff_16361_1‐p2350469’ (Data S8). The significant associated SNP is indicated by a red dot. Grey dots represent surrounding nonsignificant markers in the region. Please note that the FarmCPU GWAS method, which iteratively uses fixed‐ and random‐effects models and pseudo‐QTN as covariates, results in a different appearance of the Manhattan plots. Significant associations are illustrated by ‘helicopters’ rather than ‘skyscrapers’. For reasons of clarity and comprehensibility, the zoom‐in of the candidate region was extended to the next flanking SNP markers (‘Bn‐A04‐p1895018’ and ‘Bn‐A04‐p2094818’). Red triangles indicate the positions of selected candidate genes (Table 2). The LD heatmap in the bottom section shows the correlations (*r* ^2^) between surrounding SNP markers. The markers ‘Bn‐scaff_16361_1‐p2350469’, ‘Bn‐scaff_16361_1‐p2354073’, ‘Bn‐scaff_16361_1‐p2400621’, ‘Bn‐scaff_16361_1‐p2401475’ and ‘Bn‐scaff_16361_1‐p2402567’ form an LD block (*r* ^2^ ≥ 0.6).

From these nine markers, a promising subset of five was selected based on the number of associations and traits for detailed analysis. Candidate genes were identified in the corresponding regions on chromosomes A04, A10, C02, C03 and C08 by an LD‐based confidence interval approach (Table 2, Figure 5 and Figure S10). Genes were selected within LD blocks (r^2^ ≥ 0.6) as exemplarily shown for candidate region 5 on chromosome C08 (Figure 5), where the significantly associated marker ‘Bn‐scaff_16361_1‐p2350469’ forms an LD block with four of its neighbouring SNPs. The block spans a region of 368 kb and contains 72 genes, of which seven were selected as putative candidates based on their annotation: the citrate synthase CSY2; the MADS‐box transcription factor SHP1; PAR2 involved in the brassinosteroid‐mediated signalling pathway; the pectinesterase PME35 implicated in cell wall modification; the bHLH transcription factor PIF5; the tetrapyrrole‐binding protein GUN4 which regulates chlorophyll synthesis and the flowering time control protein FPA also annotated to be involved in cell differentiation. In case of the absence of detectable LD, genes were selected in the 100 kb flanking regions on either side of the significant marker as suggested by Zhou *et al. *(2017a).

**Table 2:** List of candidate regions and selected candidate genes

Interval	Marker	Chr.	Pos. (bp)	LD block*	Interval start (bp)	Interval stop (bp)	Interval size (bp)	Number of genes	Number of MTAs	Traits	Selected candidates†	Arabidopsis homologue/putative function‡
1	Bn‐A04‐p2218115	A04	2 103 821	no	2 003 821	2 203 821	200 000	44	14	leaf area biovolume	Bra014695 BnaA04g02550D BnaA04g02600D BnaC05g07680D	ARR17/ two‐component response regulator WRKY55/ transcription factor, WRKY ANAC064/ transcription factor, NAC domain ANAC064/ transcription factor, NAC domain
2	Bn‐A10‐p13343454	A10	12 120 357	no	12 020 357	12 220 357	200 000	54	16	FW, DW, leaf area, biovolume, plant height	BnaA10g18330D BnaA10g18440D BnaA10g18480D BnaA10g18530D BnaA10g18590D BnaA10g18600D BnaA10g18650D	NIK1/ protein phosphorylation BZIP3/ transcription factor, basic‐leucine zipper SEPALLATA1/ transcription factor, MADS‐box RRT1/ O‐fucosyltransferase, pectin biosynthetic process RGP2/ UDP‐arabinose mutase, cell wall biogenesis COBRA‐like protein/ cell wall biogenesis LONGIFOLIA1/ regulation of cell growth
3	Bn‐scaff_16804_1‐p178142	C02	9 108 149	yes	8 497 706	10 116 820	1 619 114	148	9	DW, leaf area, biovolume, plant height, plant colour uniformity	BnaC02g11320D BnaC02g11400D BnaC02g11890D BnaC02g44440D BnaC02g44470D BnaC02g11520D BnaC02g11970D BnaC02g12210D BnaC02g12340D BnaC03g72190D	SMAX1/ hydrolase, seedling development COL5/ transcription factor, zinc finger (B‐box type) AT1G50890/ cell growth ZEP2/ transcription factor, zinc finger IAA33/ auxin‐activated signalling pathway AIL5/ transcription factor, postembryonic development AT5G56960/ transcription factor, bHLH GULLO4/ oxidoreductase activity, cell wall biogenesis EXP14/ alpha‐expansion, cell growth CRK/ CDPK‐related kinase, ABA‐activated signalling pathway
4	Bn‐scaff_21312_1‐p895326	C03	11 220 963	no	11 120 963	11 320 963	200 000	43	16	DW, leaf area, biovolume	BnaC03g18580D BnaC03g18800D	IAA13/ auxin‐activated signalling pathway SPL3/ transcription factor, SBP‐box
5	Bn‐scaff_16361_1‐p2350469	C08	40 120 568	yes	39 843 456	40 211 817	368 361	72	14	leaf area, biovolume	BnaC08g29460D BnaC08g29530D BnaC08g29560D BnaCnng47940D BnaC08g29580D BnaC08g48840D BnaC08g29740D	CSY2/ citrate synthase SHP1/ transcription factor, MADS‐box PAR2/ brassinosteroid‐mediated signalling pathway PME35/ pectinesterase, cell wall modification PIF5/ transcription factor, bHLH GUN4/ tetrapyrrole‐binding, chlorophyll biosynthetic process FPA/ flowering time control, cell differentiation

* In case of the absence of an LD block, flanking 100 kb regions on either side of the associated marker were screened for candidate genes.

† Best match using BLAST of transcript sequences to the *B. napus* Darmor‐*bzh* v.4.1, the concatenated *Brassica* AC and the *A. thaliana* TAIR10 transcriptomes; a full list of de novo annotated transcripts within the five intervals, BLAST results, descriptions and functional annotations (BLAST2GO) is available in Data S5.

‡ Closest homologue in Arabidopsis thaliana; putative function (selection) obtained from the Brassica (BRAD) and the (TAIR) databases.

A comprehensive list of all thus identified candidate genes for all evaluated traits can be found in Data S5. Among the 361 genes, 30 genes were selected as particularly interesting candidates based on their annotation and gene ontology (GO). Nine of these genes have annotations related to meristem development and cell growth, including S*epallata1* (BnaA10g18480D), *Longifolia1* (BnaA10g18650D), *Squamosa promoter binding 4* (BnaC03g18800D) and S*hatterproof1* (BnaC08g29530D). Several other genes are putatively involved in flowering time or cell wall biogenesis and modification, or were annotated as transcription factors. Although candidate genes need to be further analysed and validated in follow‐up studies involving temporally and spatially resolved assessment of gene activity, the described dynamic QTL represent a well exploitable resource to deepen our knowledge of early plant growth and biomass accumulation.

Further examination of the associated markers of the five candidate regions revealed that the allele distribution differs between the three breeding pools: For example, for ‘Bn‐A04‐p2218115’, the minor allele is underrepresented in breeding pool 2 and completely absent in breeding pool 3. In contrast, the minor allele of ‘Bn‐scaff_21312_1‐p895326’ is nearly absent in breeding pool 1 and 2, but although only present in the heterozygous state, is highly overrepresented in breeding pool 3. The introgression of beneficial alleles underlying dynamic QTL, absent or underrepresented in conventional breeding pools, on the one side, as well as selection and stacking of beneficial alleles on the other side might help to enhance genetic gain for complex traits towards further improvement of growth performance in canola breeding. Moreover, it broadens the selection basis by introducing the factor temporal dynamics, facilitating marker‐assisted selection to breed high vigour cultivars.

## Author contributions

TA, RCM and DK designed the experiments. AA provided seed material. DK performed the experiments and analysed the data. FG and CRW analysed and provided genotype data. BS and RJS provided the reference assembly and transcript annotations. DK wrote the manuscript. RJS, RCM and TA managed the project, advised on interpretation and obtained the funding. All authors read and edited the manuscript.

## Supporting information


**Figure S1** Example for acquired raw image data.
**Figure S2** Overview of phenotypic data.
**Figure S3** Biomass distribution and correlation with image‐derived traits.
**Figure S4** Heritability of phenotypic traits.
**Figure S5** LD‐decay in the A and C subgenomes.
**Figure S6** Phenotypic variance explained (PVE%) by detected MTAs.
**Figure S7** Allele effects of dynamic associations.
**Figure S8** Allele effects of dynamic associations for relative growth rates.
**Figure S9** Population structure analysis.
**Figure S10** Manhattan plots for representative associations in the candidate regions with selected candidate genes and correlations between markers.
**Data S1** List of canola lines utilized in this study.
**Data S2** Overview of experimental design.
**Data S3** Phenotypic data (BLUEs), heritabilities and coefficients of variation.
**Data S4** Genotype dataset (SNP and CNV markers).
**Data S5** List of genes in candidate regions.
**Data S6** Correlations of FW, DW and biomass‐related traits.
**Data S7** Pairwise LD matrices for all chromosomes.
**Data S8** List of all associations detected during the cultivation from 6 to 28 DAS.
