# Supplementary material for: Strong temporal dynamics of QTL action on plant growth progression revealed through high‐throughput phenotyping in canola
Source: Plant Biotechnol J. 2019 Jun 12;18(1):68–82. doi: 10.1111/pbi.13171 (PMC6920335; doi:10.1111/pbi.13171)
Supplement: Supplementary file 1 — Figure S1 Example for acquired raw image data. Figure S2 Overview of phenotypic data. Figure S3 Biomass distribution and correlation with image‐derived traits. Figure S4 Heritability of phenotypic traits. Figure S5 LD‐decay in the A and C subgenomes. Figure S6 Phenotypic variance explained (PVE%) by detected MTAs. Figure S7 Allele effects of dynamic associations. Figure S8 Allele effects of dynamic associations for relative growth rates. Figure S9 Population structure analysis. Figure S10 Manhattan plots for representative associations in the candidate regions with selected candidate genes and correlations between markers. Data S1 List of canola lines utilized in this study. Data S2 Overview of experimental design. Data S3 Phenotypic data (BLUEs), heritabilities and coefficients of variation. Data S4 Genotype dataset (SNP and CNV markers). Data S5 List of genes in candidate regions. Data S6 Correlations of FW, DW and biomass‐related traits. Data S7 Pairwise LD matrices for all chromosomes. Data S8 List of all associations detected during the cultivation from 6 to 28 DAS. [file PBI-18-68-s001.docx]

**SUPPORTING INFORMATION**

**Strong temporal dynamics of QTL action on plant growth progression revealed through high-throughput phenotyping in canola**

Figure S1. Example for acquired raw image data

Figure S2. Overview of phenotypic data

Figure S3. Biomass distribution and correlation with image-derived traits

Figure S4. Heritability of phenotypic traits

Figure S5. LD-decay in the A and C subgenomes

Figure S6. Phenotypic variance explained (PVE%) by detected MTAs

Figure S7. Allele effects of dynamic associations

Figure S8. Allele effects of dynamic associations for relative growth rates

Figure S9. Population structure analysis

Figure S10. Manhattan plots for representative associations in the candidate regions with selected candidate genes and correlations between markers

Data S1. List of canola lines utilized in this study

Data S2. Overview of experimental design

Data S3. Phenotypic data (BLUEs), heritabilities and coefficients of variation

Data S4. Genotype dataset (SNP and CNV markers)

Data S5. List of genes in candidate regions

Data S6. Correlations of FW, DW and biomass-related traits

Data S7. Pairwise LD matrices for all chromosomes

Data S8. List of all associations detected during the cultivation from 6 to 28 DAS

Link to Data S1-S8: <http://dx.doi.org/10.5447/IPK/2019/10>

**Figure S1. Example for acquired raw image data**

**
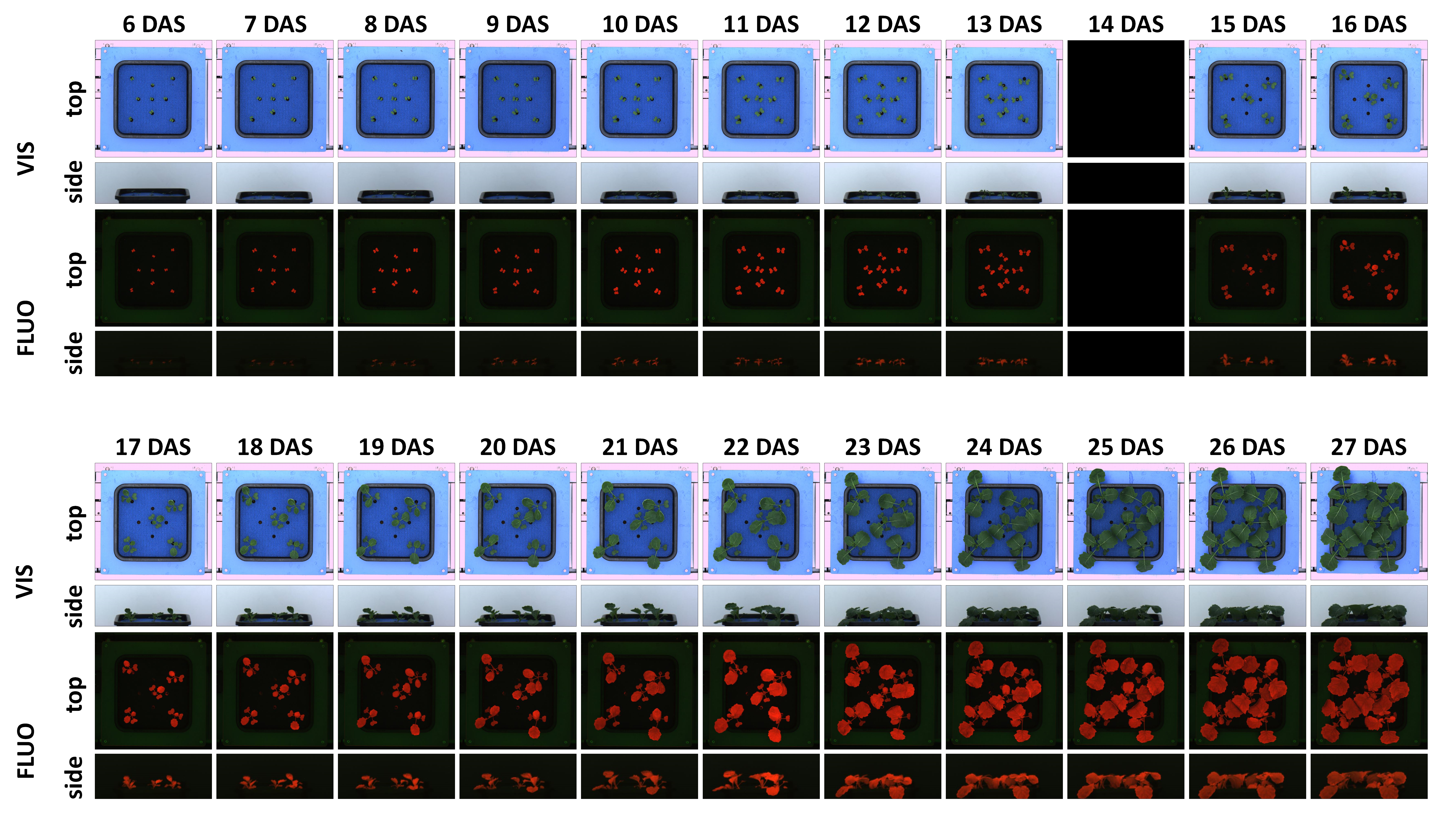
**

**Figure S1. Example for acquired raw image data**

Carrier 1087 (Experiment III, Pollinator 083) is shown as an example for the acquired raw imaging data. Plants were phenotyped daily with two camera systems. Visible light (VIS) and static fluorescence (FLUO) image data were recorded as top and side view from 6 to 27 DAS. At 14 DAS no imaging was performed due to sampling of early shoot material for molecular and biochemical analyses.

**Figure S2. Overview of phenotypic data**

**
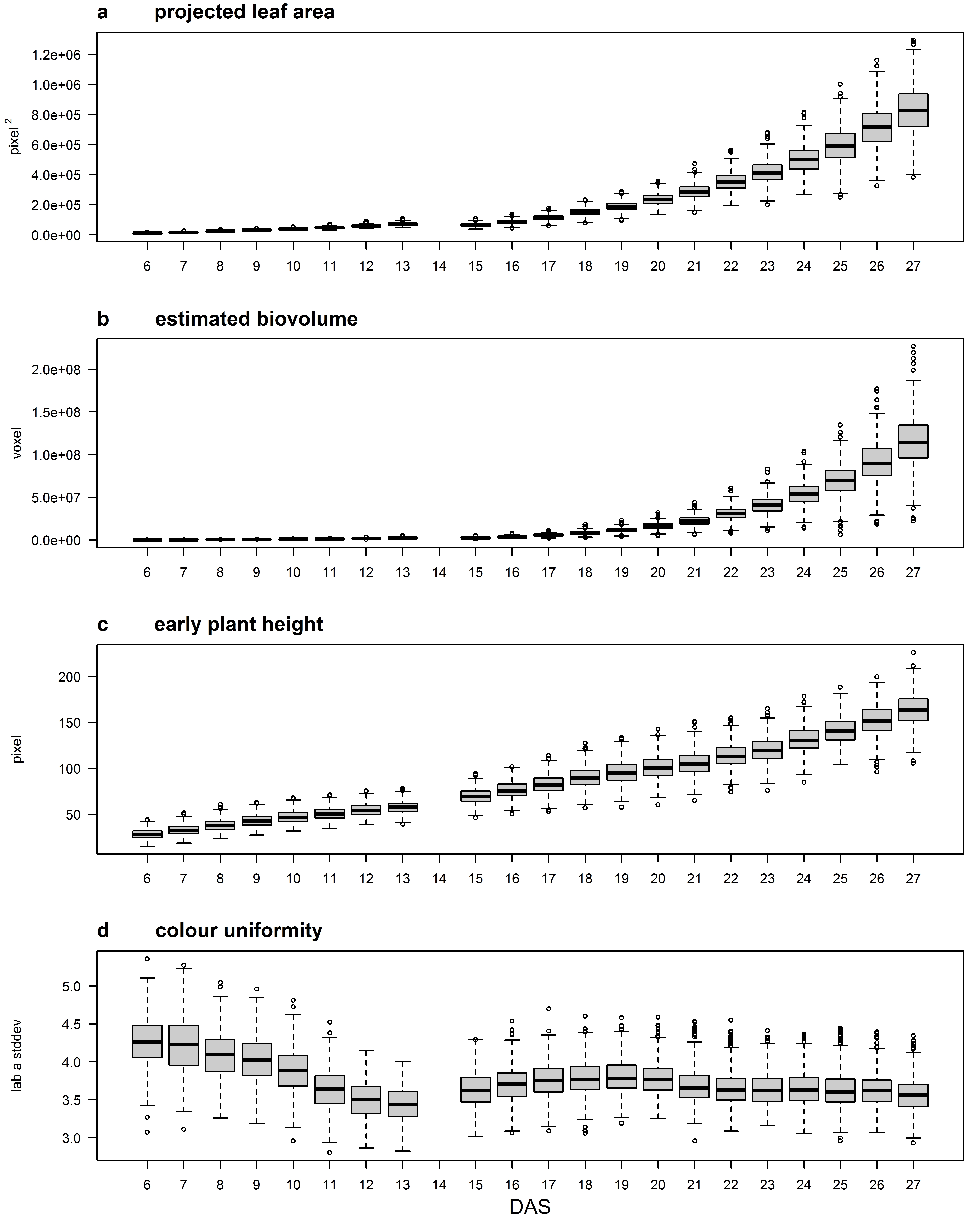
**

**
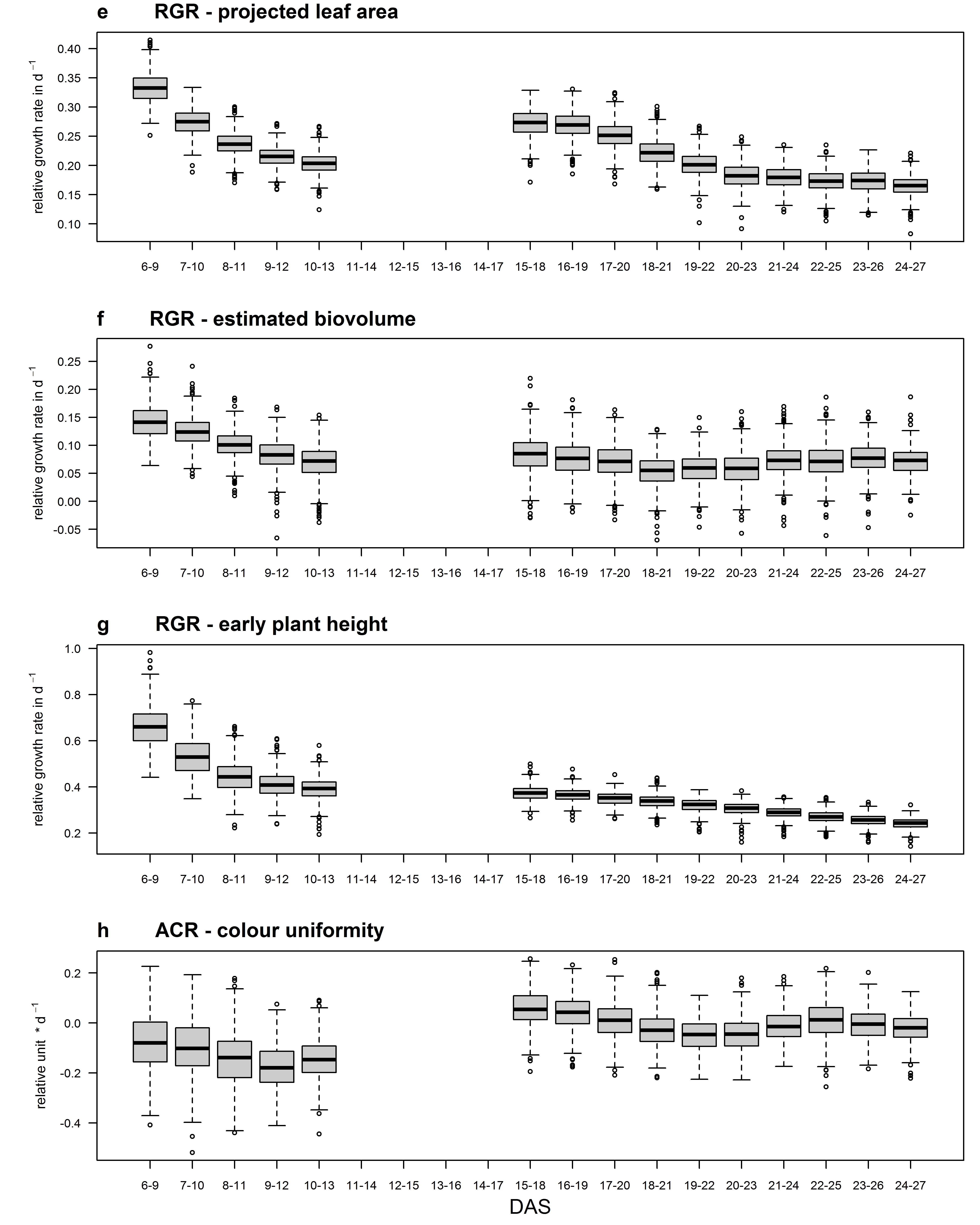
**

**Figure S2. Overview of phenotypic data**

Boxplots represent BLUEs across experiments of **a** projected leaf area, **b** estimated biovolume, **c** early plant height and **d** plant colour uniformity. Colour uniformity is given as the standard deviation of the a-values in the L*a*b* colour space of the plant pixels. The lower this value, the more uniform is the plant colour. BLUEs for relative growth rates (RGRs) and absolute change rates (ACRs) were calculated over three days and are shown as **e** RGRs based on projected leaf area, **f** RGRs based on estimated biovolume, **g** RGRs based on early plant height and **h** ACRs on colour uniformity.

**Figure S3. Biomass distribution and correlation with image-derived traits**

**
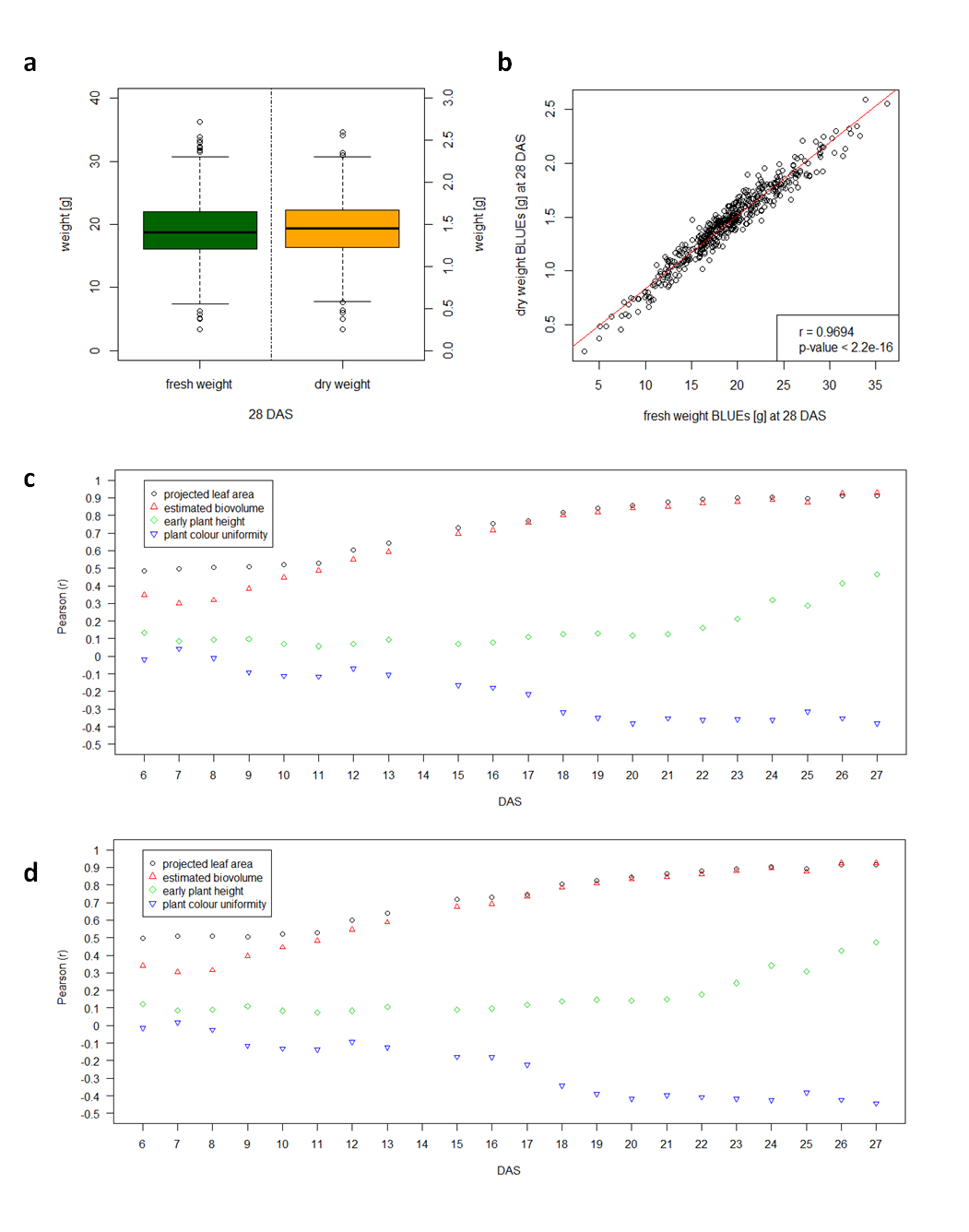
**

**Figure S3. Biomass distribution and correlation with image-derived traits**

**a** End-point biomass data as fresh weight (FW, in green) and dry weight (DW, in orange) were manually determined at 28 DAS. BLUEs for both traits were estimated based on 15 individual plants grown in an incomplete randomized block design over four experiments. Data are displayed as boxplots. **b** Pearson correlation of fresh weight (FW) and dry weight (DW)
**c** Pearson correlations of fresh weight (FW) with image-derived traits. **d** Pearson correlations of dry weight (DW) with image-derived traits.

**Figure S4. Heritability of phenotypic traits**

**
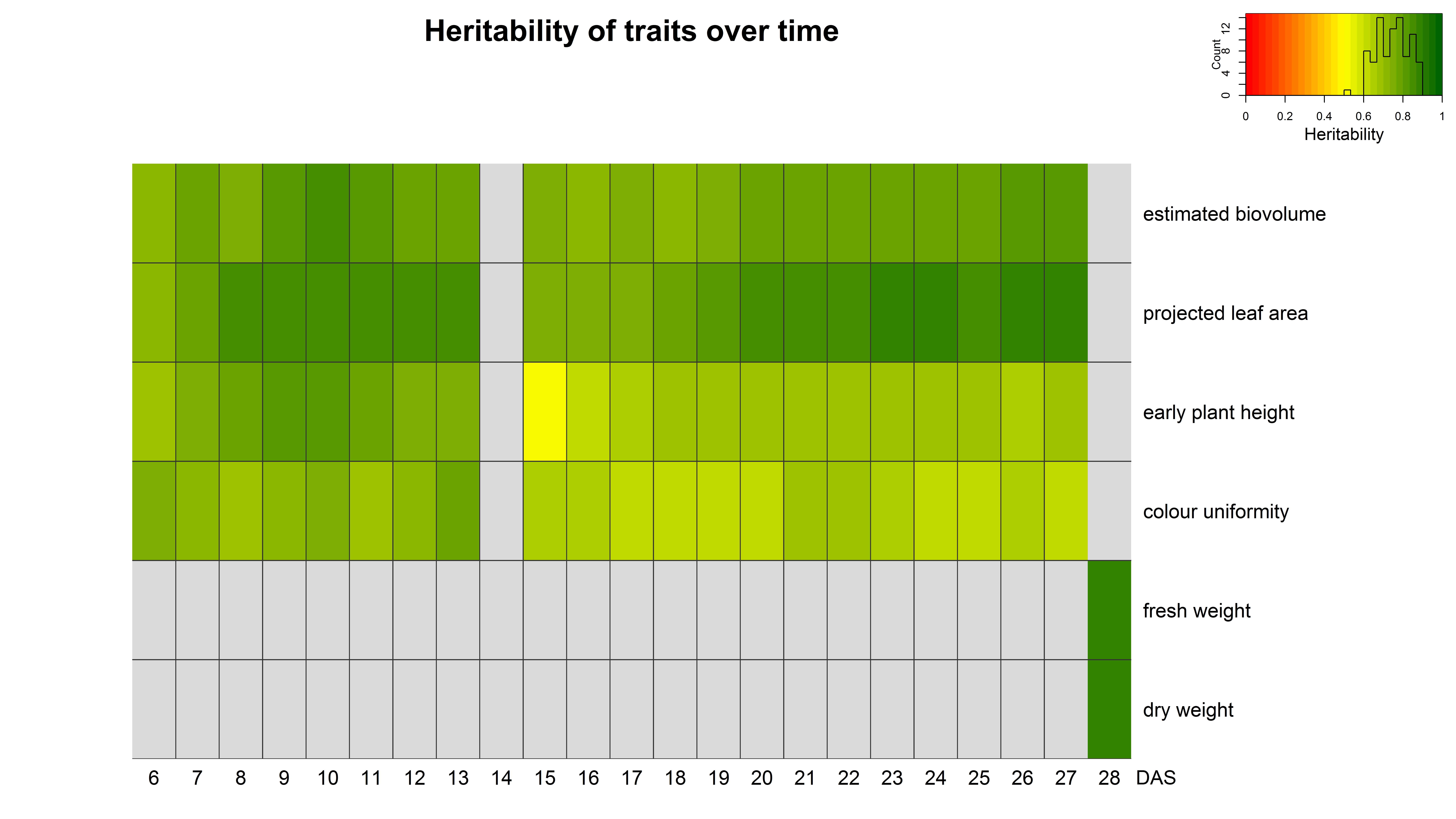
**

**Figure S4. Heritability of phenotypic traits**

Broad-sense heritability values (H^2^) of the four image-derived traits (estimated biovolume, projected leaf area, early plant height, colour uniformity) and end-point biomass are shown as heatmap over time (6 to 28 DAS). H^2^ for end-point biomass was estimated based on 15 individual plants grown in an incomplete randomized block design. H^2^ values for the four image-derived traits were estimated for each time point individually, based on three replicates (carriers). Grey colour indicates missing values.

**Figure S5. LD-decay in the A and C subgenomes**


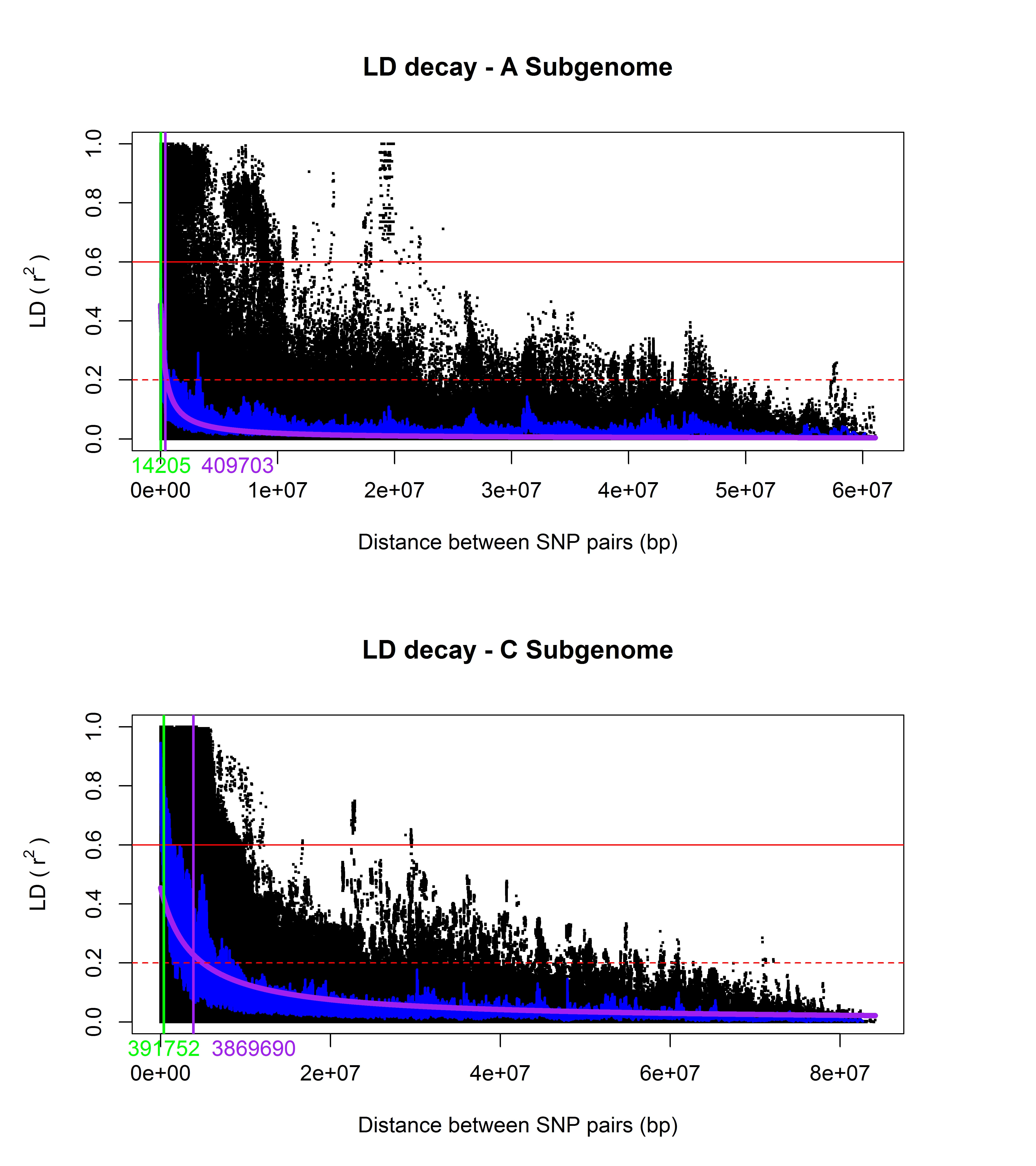


**Figure S5. LD-decay in the A and C subgenomes**

Pairwise marker linkage disequilibrium (LD) was calculated as r^2^ values from the SNP data and plotted against the physical marker distances on the *B. napus* A and C subgenomes, separately. The blue line represents a rolling mean of LD of 100 markers. The purple line shows the LD decay calculated according to Hill and Weir (1988) using a non-linear model. The horizontal solid and dashed lines indicate an LD of 0.6 and 0.2, respectively. The vertical green line indicate the distance where the rolling mean drops below a LD value of 0.6, and the purple line the half-decay, respectively. Both the half-decay position and the position at LD 0.6 were substantially higher in the C subgenome than in the A subgenome.

**Figure S6. Phenotypic variance explained (PVE%) by detected MTAs**

**
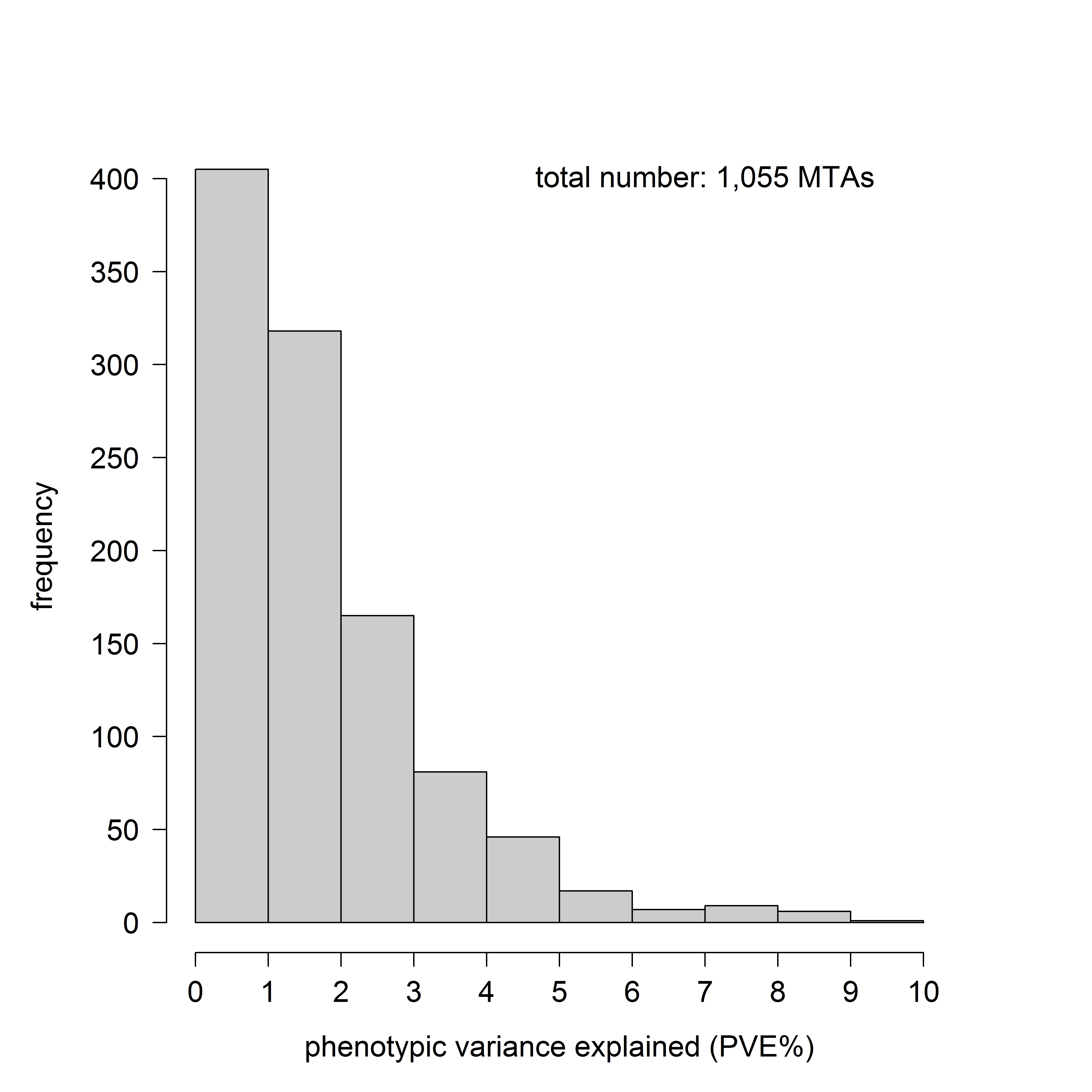
**

**Figure S6. Phenotypic variance explained (PVE%) by detected MTAs**

Histogram of phenotypic variance explained (PVE%) of all 1,055 marker-trait associations (MTAs) with a *p*-value _(FDR)_ ≤ 0.1 detected in this study for projected leaf area (n=200), estimated biovolume (n=191), early plant height (n=182), colour uniformity (n=192), end-point fresh (n=13) and dry weight (n=9), as well as relative growth rates for area (n=76), volume (n=100) and height (n=73), and absolute change rates for colour uniformity (n=19). On average markers explained 1.72 PVE%. Individual markers explained up to 9.05 PVE% of particular traits. A comprehensive list of all MTAs is provided as Data S8.

**Figure S7. Allele effects of dynamic associations**


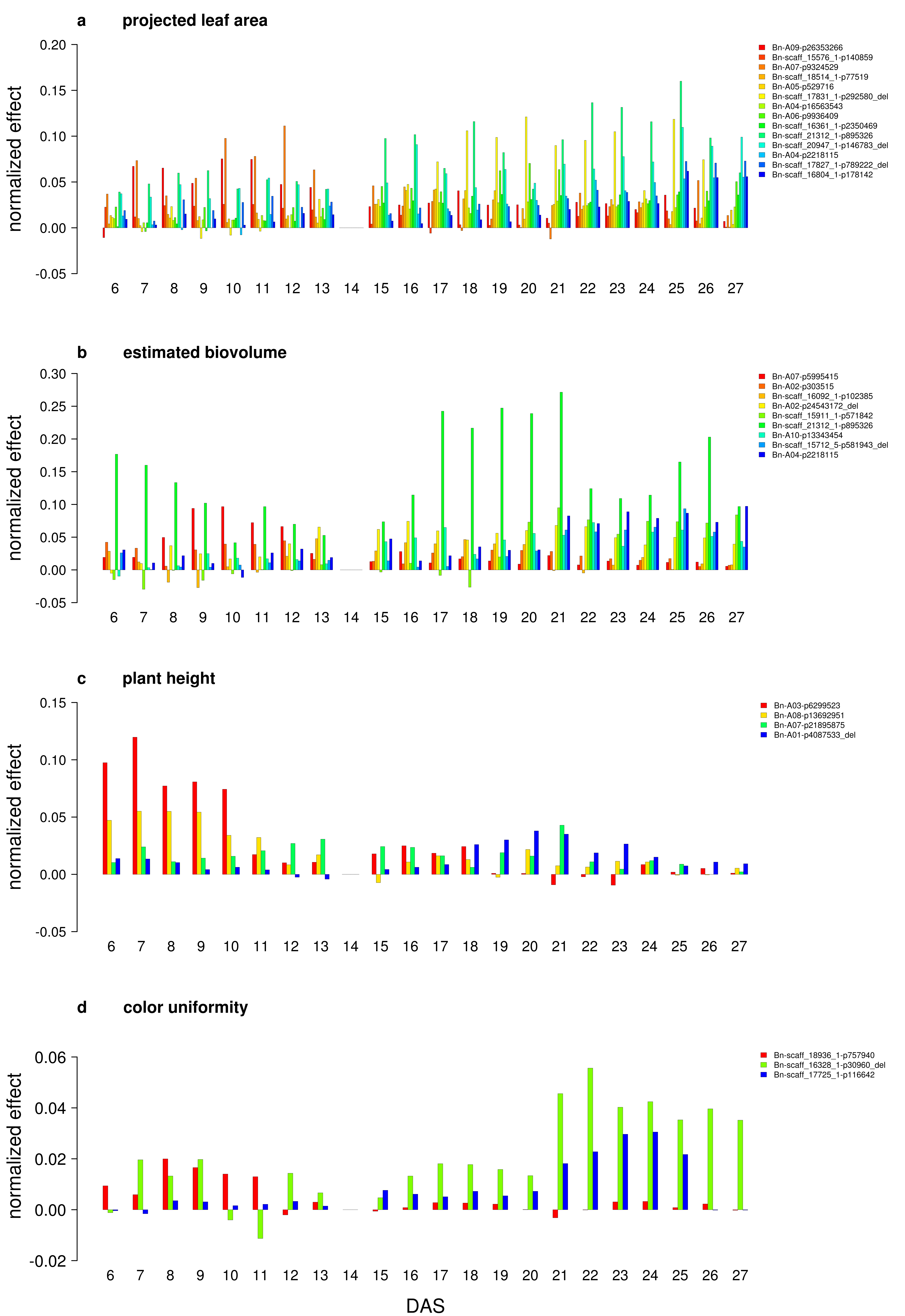


**Figure S7. Allele effects of dynamic associations**

GWAS was performed on BLUEs of **a** projected leaf area, **b** estimated biovolume, **c** early plant height and **d** plant colour uniformity in R / package {FarmCPU}. Different colours indicate markers with *p*-value _(FDR)_ ≤0.1 at three consecutive days, with the colour gradient corresponding to the temporal pattern. DAS denotes days after sowing. BLUEs were estimated using three replicates (carriers) with nine and five plants for 6 to 13 DAS and 15 to 27 DAS, respectively. No data was recorded at 14 DAS due to sampling of shoot material. Allele effects were normalized by dividing the effects by the median of the phenotypic trait of each day. For simplification predominantly negative allele effects were inversed.

**Figure S8. Allele effects of dynamic associations for relative growth rates**

**
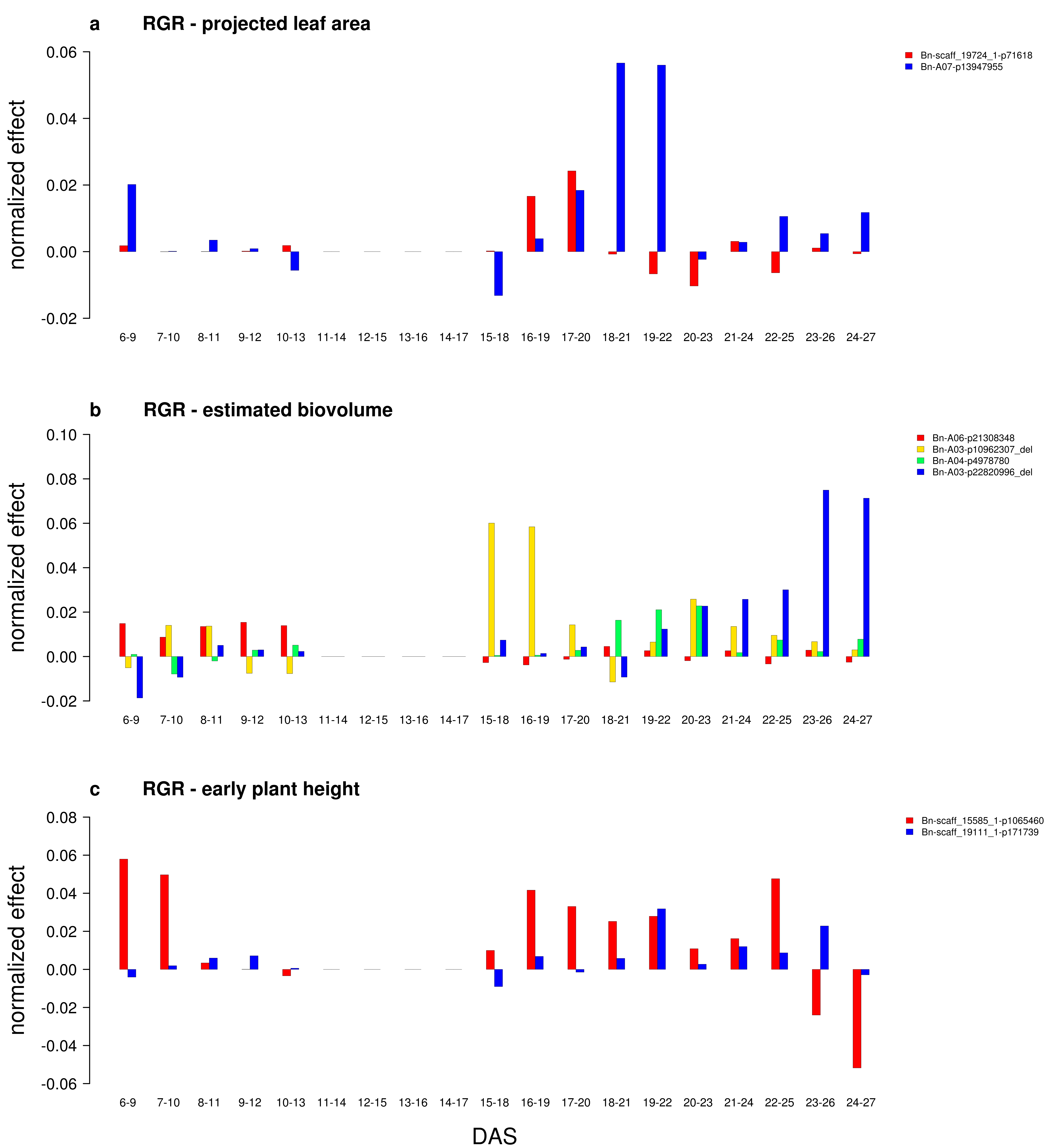
**

**Figure S8. Allele effects of dynamic associations for relative growth rates**

GWAS was performed on BLUEs of **a** RGR - projected leaf area, **b** RGR - estimated biovolume and **c** RGR - early plant height in R / package {FarmCPU}. Different colours indicate markers with *p*-value _(FDR)_ ≤0.1 at two consecutive intervals. DAS denotes days after sowing. BLUEs were estimated using three replicates (carriers) with nine and five plants for 6 to 13 DAS and 15 to 27 DAS, respectively. No data was recorded at 14 DAS due to sampling of shoot material. Allele effects were normalized by dividing the effects by the median of the phenotypic trait of each day. For simplification predominantly negative allele effects were inversed

**Figure S9. Population structure analysis**

**
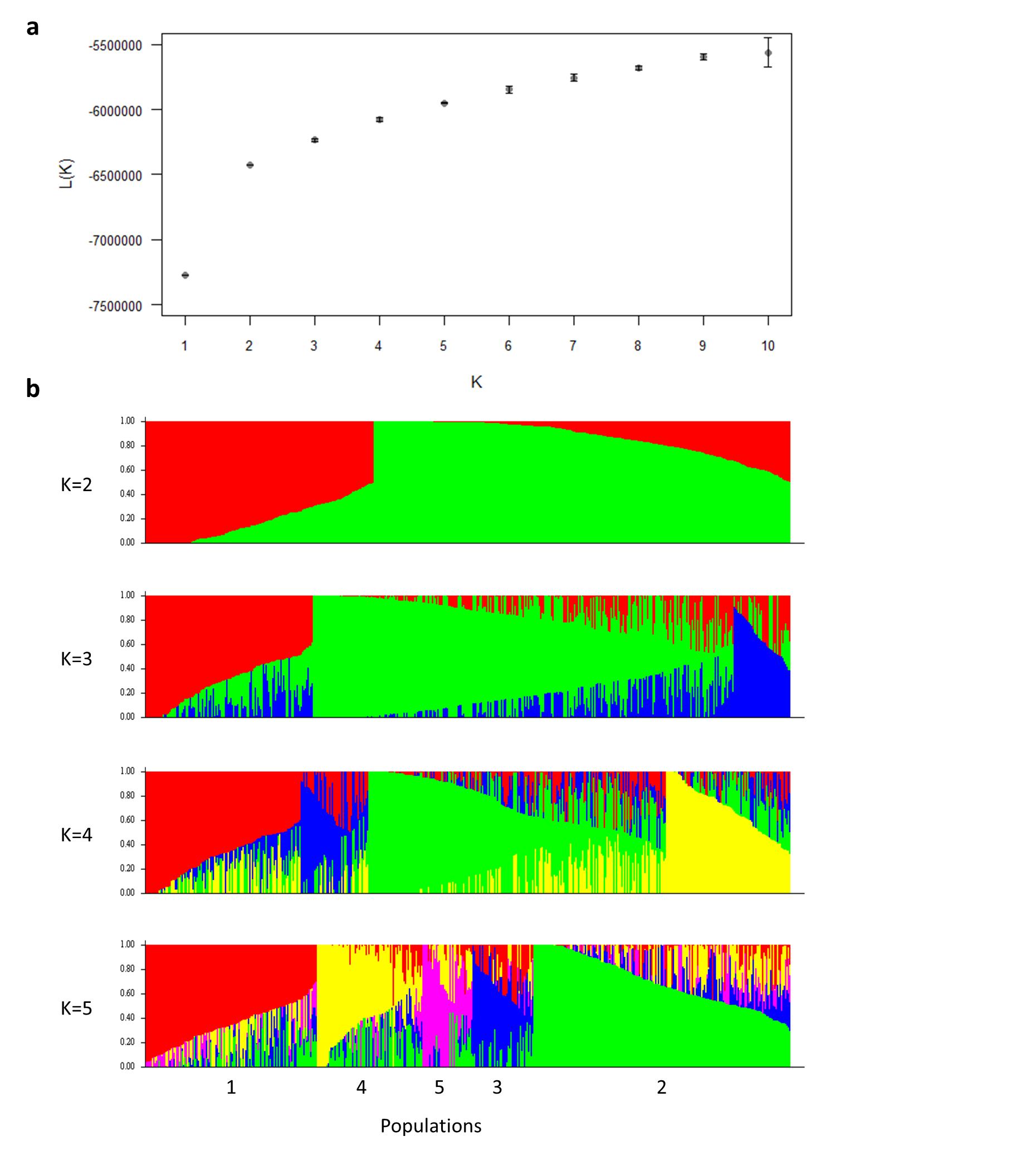
**

**Figure S9. Population structure analysis**

Population structure for all 477 *Brassica napus* lines subjected to GWAS was analysed using the programme STRUCTURE, version 2.3.4 (Pritchard *et al.*, 2000). Population clustering for *K=* 1 to 10 was performed using the ‘admixture’ model with a burn-in period of 10.000, 10.000 MCMC replications and three iterations per K. The lambda parameter was set to λ= 0.304. **a** Mean Ln probability [ L(K) ] and standard deviation for K= 1 to 10. **b** Plots for *K=* 2 to 5. Lines were sorted by their ancestry vector (Q). Each genotype is represented by a thin vertical line. Each colour represents a population, and the colour of individual genotypes represents their proportional membership in the different populations. Shown are population structures for populations 1 to 5. Populations: red, pop1; green, pop2; blue, pop3; yellow, pop4; pink, pop5.

**Figure S10. Manhattan plots for representative associations in the candidate regions with selected candidate genes and correlations between markers**

**
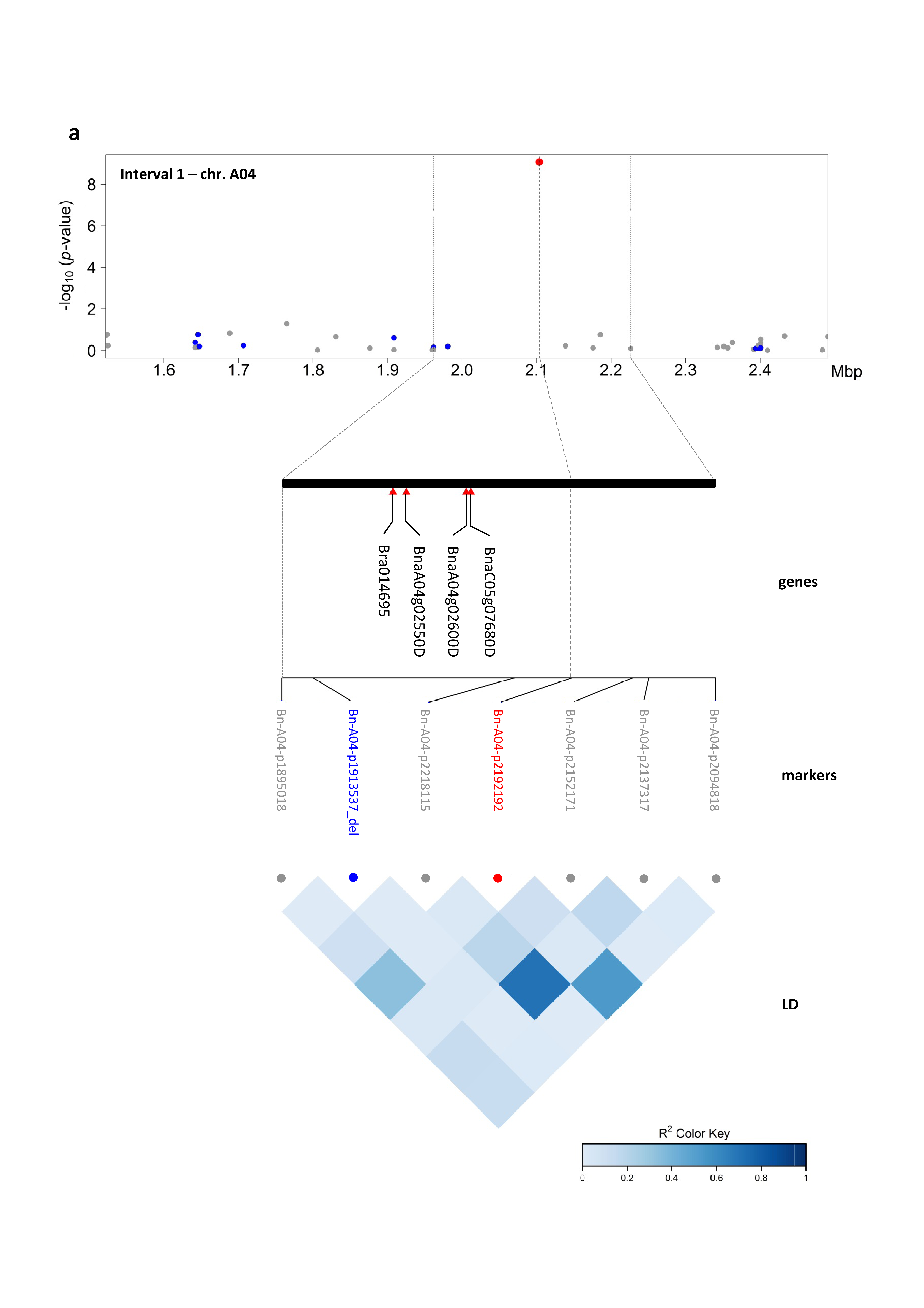
**


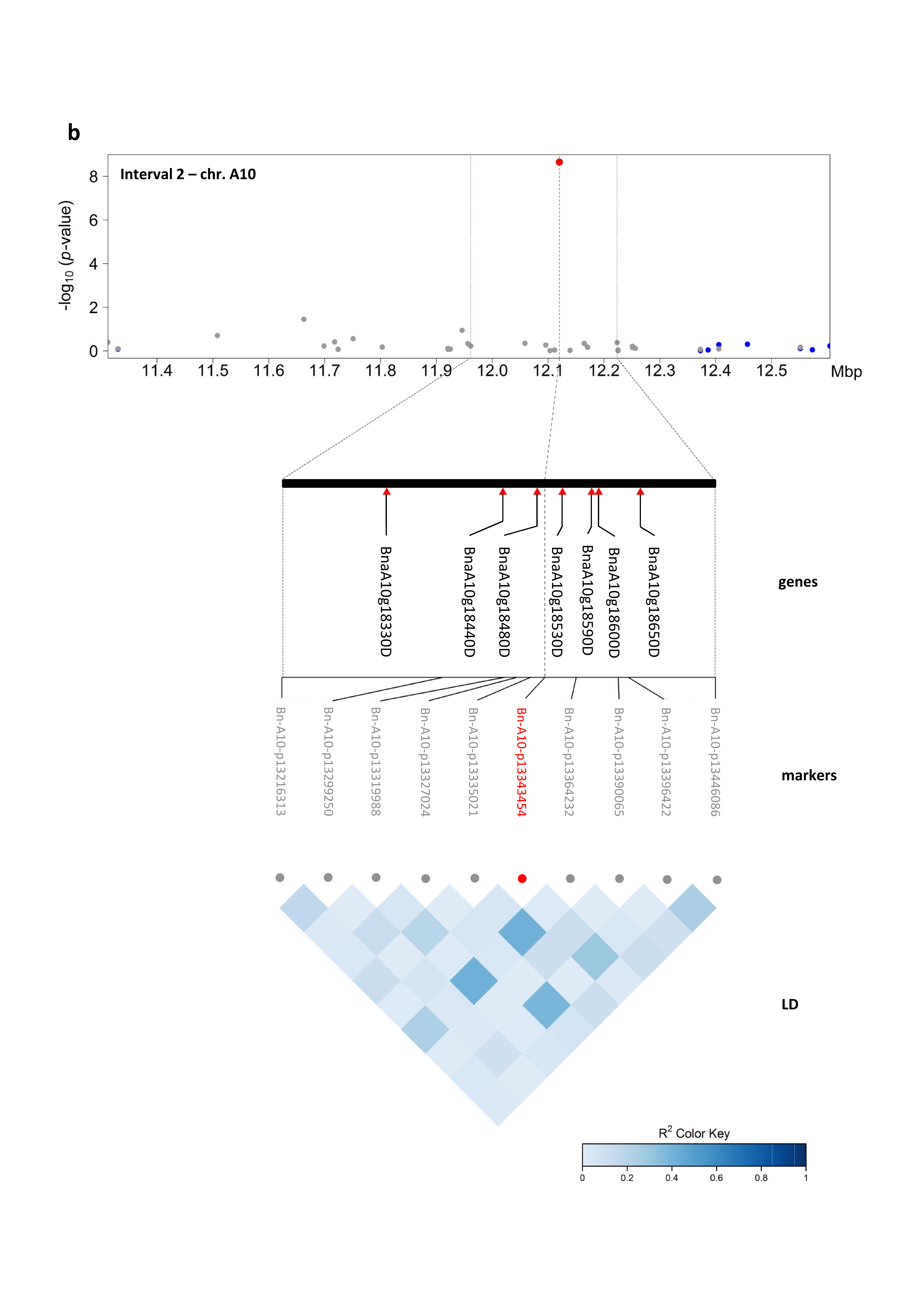


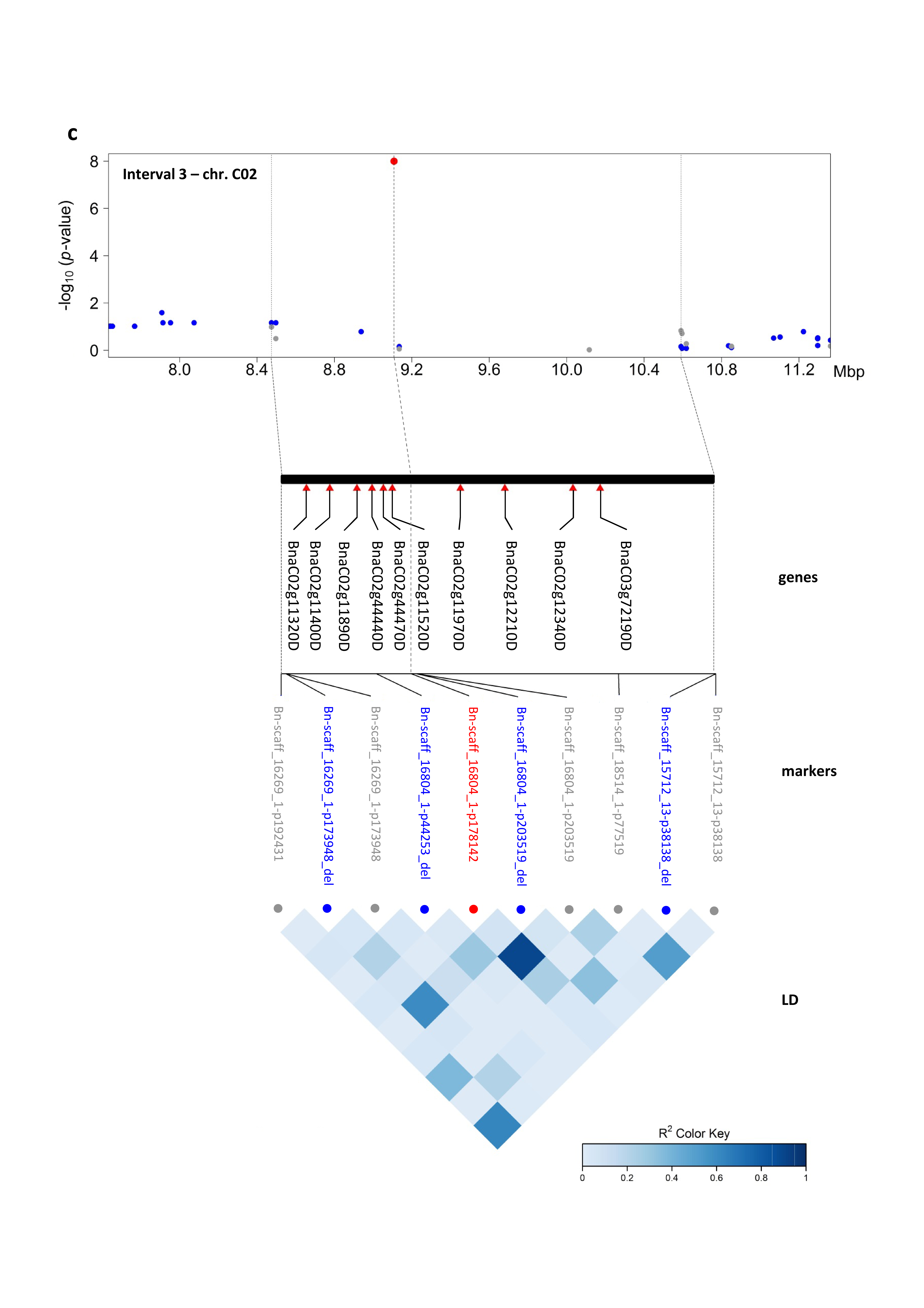


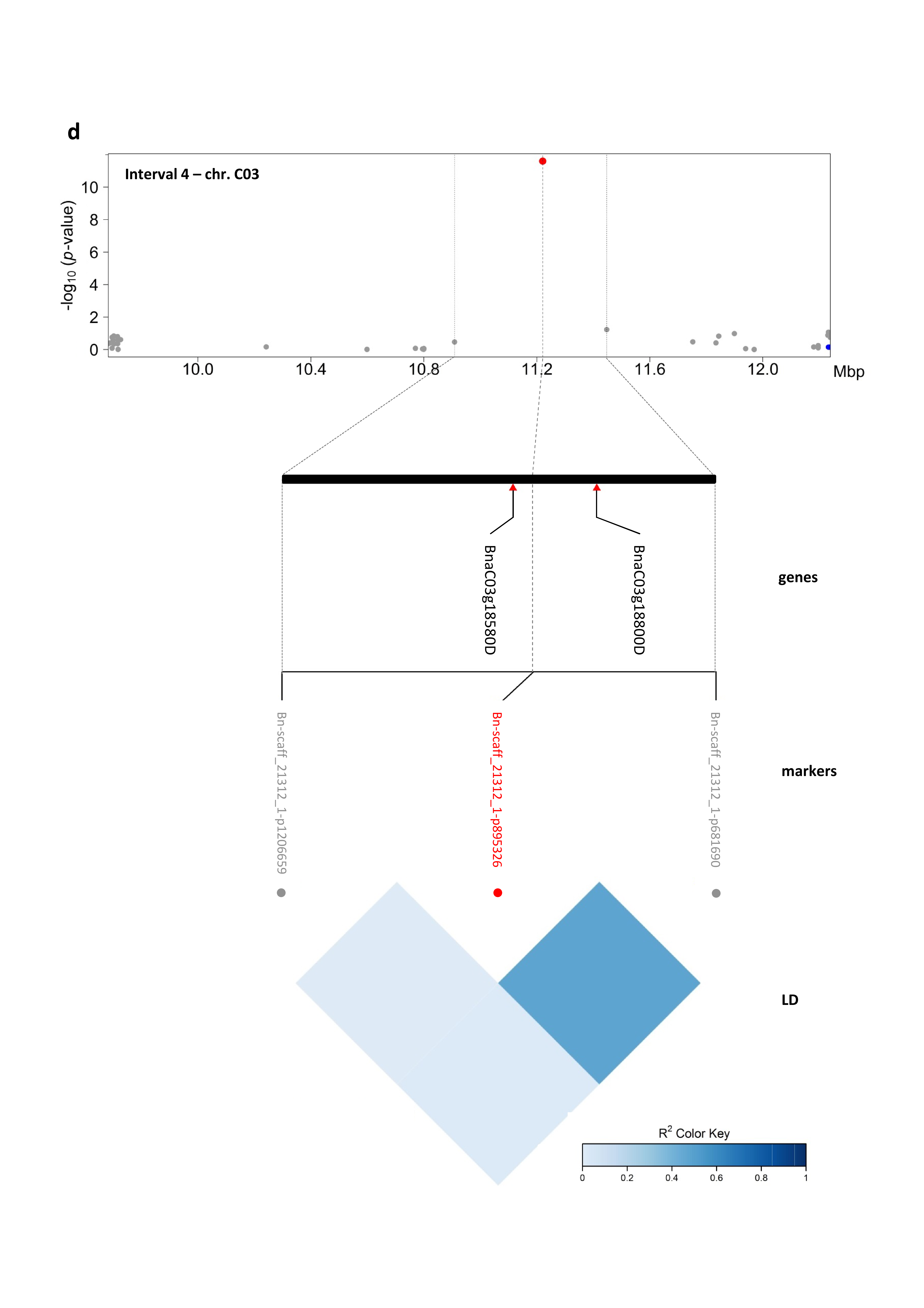


**Figure S10. Manhattan plots for representative associations in the candidate regions with selected candidate genes and correlations between markers**

The Manhattan plots describe genome-wide marker-trait associations for four of the five candidate regions (Table 2). **a** Interval 1 on chromosome A04. The trait ‘estimated biovolume at 27 DAS’ is shown as a representative trait for the 14 traits associated with the marker ‘Bn-A04-p2218115’. **b** Interval 2 on chromosome A10. The trait ‘estimated biovolume at 22 DAS’ is shown as a representative trait for the 16 traits associated with the marker ‘Bn-A10-p13343454’. **c** Interval 3 on chromosome C02. The trait ‘colour uniformity at 27 DAS’ is shown as a representative trait for the 9 traits associated with the marker ‘Bn-scaff_16804_1-p178142’. The CNV markers were usually not in LD with the SNP markers and therefore disrupt the structure of the LD blocks. Hence, the two SNP markers ‘Bn-scaff_16804_1-p178142’ and ‘Bn-scaff_16804_1-p203519’ should be regarded as LD block. **d** Interval 4 on chromosome C03. The trait ‘estimated biovolume at 21 DAS’ is shown as a representative trait for the 16 traits associated with the marker ‘Bn-scaff_21312_1-p895326’. The significantly associated SNPs are indicated by red dots. Grey and blue dots represent surrounding non-significant SNP and CNV markers, respectively. Please note that the FarmCPU GWAS method, which iteratively uses fixed and random effect models and pseudo QTN as covariates, results in a different appearance of the Manhattan plots. Significant associations are illustrated by ‘helicopters’ rather than ‘skyscrapers’. For reasons of clarity and comprehensibility, the zoom-in of the candidate regions was extended to the next flanking SNP markers. Red triangles indicate the positions of selected candidate genes (Table 2). The LD heatmaps in the bottom sections show the correlations (r^2^) between surrounding SNP markers.
